# Exploring the m^5^C epitranscriptome of mRNAs in breast cancer cells through genome engineering and long-read sequencing approaches

**DOI:** 10.1007/s10142-025-01648-4

**Published:** 2025-06-25

**Authors:** Konstantina Athanasopoulou, Panagiotis G. Adamopoulos, Panagiotis Tsiakanikas, Andreas Scorilas

**Affiliations:** Department of Biochemistry and Molecular Biology, Faculty of Biology, National and Kapodistrian University of Athens, Panepistimiopolis, Athens, 15701 Greece

**Keywords:** Epitranscriptomics, 5-methylcytosine, Nanopore sequencing, RNA biology, Breast cancer, mRNA modifications, Gene expression, Post-transcriptional regulation

## Abstract

Epitranscriptomics has emerged as a rapidly evolving field that focused on studying post-transcriptional RNA modifications and their role in spatiotemporal regulation of gene expression. N6-methyladenosine (m^6^A) and 5-methylcytosine (m^5^C) represent the most extensively studied modifications on mRNAs. These reversible modifications, mediated by ‘writer,’ ‘eraser,’ and ‘reader’ proteins, dynamically fine-tune mRNA stability, splicing, and translation. Growing evidence links their dysregulation to pathological states, including cancer progression and metastasis, where their aberrant deposition on oncogenes or tumor suppressors alters cellular signaling and therapeutic responses. In the current study, we present a detailed analysis of the m^5^C epitranscriptomic landscape across distinct breast cancer molecular subtypes. Using CRISPR/Cas9, we confirm NSUN2 as a key m^5^C writer in human mRNAs. NSUN2 loss was validated by targeted sequencing and Western blotting. Furthermore, we demonstrate the regulatory effects of NSUN2 on its canonical mRNA targets, revealing its critical role in maintaining proper gene expression networks. Our findings strongly suggest that additional m^5^C writers contribute to m^5^C methylation machinery. Additionally, we assessed the functional impact of NSUN2 depletion on mRNAs harboring m^5^C sites using mRNA stability assays. Furthermore, our analysis revealed distinct m^5^C methylation patterns among breast cancer subtypes, highlighting unique m^5^C signatures associated with the disease. Notably, we identified specific hypomethylated and hypermethylated m^5^C sites in each breast cancer cell line, representing a universal m^5^C methylation signature for breast cancer. Our study constitutes the first comprehensive m^5^C epitranscriptomic atlas in human breast cancer and paves the way for future research aimed at developing targeted therapeutic interventions that leverage the m^5^C methylation landscape.

**Supplementary Information:**

The online version contains supplementary material available at 10.1007/s10142-025-01648-4.

## Introduction

The integration of ‘multi-omics’ approaches is essential in unraveling the complex cellular mechanisms that drive tumorigenesis. Epitranscriptomics study the dynamic interplay of RNA modifications and their regulatory roles across genomic, transcriptomic, and proteomic layers (Saletore et al. 2012). While N6-methyladenosine (m^6^A) is under the spotlight of the current epitranscriptomic research as the most prevalent mRNA modification (Wang et al. 2022), the present work shifts the focus to another pivotal modification, 5-methylcytosine (m^5^C). Epitranscriptomic dysregulation—such as aberrant activity of ‘writer’ or ‘eraser’ enzymes— rewires signaling pathways, driving pathological states such as cancer progression, metastasis, and therapy resistance (Song et al. 2022). Transcriptome-wide analyses are crucial in defining m^5^C epitransciptomic landscape, which is a foundational step in unraveling its regulatory roles in cancer biology. Besides the identification of context-specific m^5^C deposition patterns on mRNAs, functional studies are critical to assess the regulatory impact of these modifications on RNA stability, translation efficiency, and mRNA-protein interactions. Multiple studies suggest that various methyltransferases, including members of the NOL1/NOP2/SUN domain (NSUN) family that constitute established m^5^C writers in RNAs, are associated with migration, invasion and metastasis in breast cancer, whereas the high expression of the m^5^C reader, *ALYREF*, has been correlated with poor survival in patients suffering from breast cancer (Klec et al. 2022; Okamoto et al. 2012). A profound hypothesis related to this observation is that these biological outcomes are potentially mediated by m^5^C. Investigating the transcriptome-wide distribution of m^5^C modifications and associating these patterns with defects in m^5^C regulatory proteins will reveal mechanistic links between epitranscriptomic dysregulation and cancer progression, facilitating the development of novel therapeutic strategies in precision oncology (Wei et al. 2020).

Conventional methods, such as chemical approaches employing dimethyl sulfate (DMS) probing and selective cleavage with RNase H, have already been utilized to identify m^5^C sites. During treatment with sodium bisulfite, unmethylated cytosines undergo deamination hence converted to uracil, while methylated cytosines remain unaffected (Schaefer et al. 2009). While bisulfite-based methods achieve single-nucleotide resolution by identifying non-converted cytosines in cDNA, they are prone to false positives due to their inability to discern between chemical modifications that exert a similar protective role on cytosines during bisulfite-mediated conversion such as 5-hydroxymethylcytidine, hm^5^C. Mass spectrometry constitutes a well-established alternative method to precisely assess m^5^C abundance in RNA samples, however it cannot provide with transcriptome-wide mapping or resolve the precise localization of m^5^C modifications on individual RNA molecules (Lauman and Garcia 2020; Zhang et al. 2022). The previously described drawbacks have been alleviated with advances in high-throughput sequencing technologies, including RNA-seq, which enable accurate mapping of RNA modifications on a transcriptome-wide scale. Next-generation sequencing (NGS) techniques, such as miCLIP-seq, utilize m^5^C-specific antibodies to immunoprecipitate, enrich and detect m^5^C-modified RNA sequences (Chen et al. 2021). While NGS is a powerful tool for studying RNA modifications, certain limitations persist across its applications. NGS-based methods, such as bisulfite RNA sequencing (bsRNA-seq), Aza-IP, and miCLIP can achieve single-nucleotide resolution for mapping m^5^C. However, these techniques require chemical treatment of the sequencing template, which has significant drawbacks, including RNA fragmentation due to bisulfite treatment and ambiguous signal interpretation caused by incomplete conversion, leading to false positives and inconsistent sequencing coverage. These drawbacks can be leveraged by immunoprecipitation-based strategies to selectively enrich modified RNAs. The utilization of specialized antibodies for each specific modification lacks precision and provides only relative positioning of m^5^C modifications. Furthermore, RNA immunoprecipitation coupled with NGS may produce false positives due to reduced antibody specificity and the impact of RNA secondary structures (Yang et al. 2017).

Recently, nanopore sequencing technology has emerged as a promising alternative to address the limitations of NGS. This approach directly identifies multiple modified nucleotides on RNAs in a single run, eliminating the need for chemical treatments or antibody-based enrichment. Direct RNA sequencing using nanopore platforms generates long reads, enabling single-nucleotide resolution in real-time. This capability allows for precise mapping of RNA modifications within full-length transcripts, while retaining their native RNA context—critical for understanding functional roles in splicing, localization, or protein interaction networks. (Leger et al. 2021; Stephenson et al. 2022; White and Hesselberth 2022). Recently, a plethora of bioinformatics tools have been designed to analyze the electrical current produced by nanopore sequencing to identify m^6^A-modified adenosines (Leger et al. 2021). Nevertheless, only a few pipelines for detecting m^5^C sites are available and therefore the utilization of nanopore datasets in the study of m^5^C distribution and profiling across the human transcriptome remains elusive (Zhang et al. 2020). The development of advanced tools for detecting RNA modifications is expected to significantly transform the field of epitranscriptomics. These tools will enable detailed characterization of RNA modifications and provide critical insights into the epitranscriptomic landscape, enhancing our understanding of its role in gene regulation.

The current study delves into the intricate world of m^5^C modification in mRNAs through the utilization of nanopore sequencing in an established panel of breast cell lines originating from major breast cancer molecular subtypes. The initial goal of our study was to provide a detailed overview of m^5^C distribution within mRNAs among the different breast cancer cell types. Furthermore, by implementing CRISPR/Cas-based genome editing (Yang and Zhang 2023), we sought to explore the impact of major writers on m^5^C epitranscriptome of mRNAs. In specific, we designed and employed a targeted CRISPR/Cas9 approach that enabled the knockout (KO) of *NSUN2* gene, which is characterized as a major m^5^C writer, to investigate the m^5^C-related consensus sequences into the human transcriptome. The functional impact of NSUN2 KO on m^5^C-mediated regulation of mRNAs metabolism was assessed through mRNA stability assays, using targets harboring NSUN2-dependent m^5^C sites. To further elucidate the role of NSUN2 in gene regulation, we examined the expression levels of its well-established canonical targets, including YBX1, IGF2BP1, DNMT2, and ALYREF in both wild-type (WT) and NSUN2-deficient (NSUN2-) cells. Our study ultimately investigates the m^5^C epitranscriptome across different molecular subtypes of breast cancer, providing novel insights into breast cancer biology and lay the foundation to develop epitranscriptomic-oriented therapeutic interventions.

## Materials and methods

### Biological material

To investigate the human m^5^C methylome of breast cancer, we used MCF-7 (Luminal A), BT-474 (Luminal B), SK-BR-3 (HER2+), BT-20 and MDA-MB-231 (Basal-like TNBC) cell lines, which represent the major molecular subtypes of breast cancer. Additionally, the breast epithelial cell line MCF-10A was utilized as a non-cancerous reference cell line, to compare the findings derived from different breast cancer molecular subtypes. All cell lines were cultured based on the handling instructions of American Type Culture Collection (ATCC).

### Total RNA extraction and mRNA selection

Total RNA isolation from each cell line was performed using the TRIzol Reagent (Ambion™, Thermo Fisher Scientific Inc.). All RNAs were appropriately diluted in the RNA Storage Solution (Ambion™) and quantified using the Qubit^®^ RNA HS Assay Kit (Invitrogen™, Thermo Fisher Scientific Inc.). Subsequently, the positive selection of the polyA + RNAs was accomplished with the Magnosphere™ UltraPure mRNA Purification Kit (Takara Bio Inc.), according to the guidelines of the manufacturer. Concentration and quality assessment was performed with capillary electrophoresis using the Agilent 2100 Bioanalyzer and an RNA 6000 Pico kit (Agilent Technologies, Foster City, CA, USA).

### Golden gate cloning and bacterial transformation

An all-in-one plasmid for CRISPR/Cas9 genome editing was obtained from Addgene (plasmid #62988). This plasmid encodes the Cas9 protein from *S. pyogenes*, includes the *pac* gene for puromycin resistance, and contains one cloning site for a sgRNA. Prior to ligation, the gRNA overhangs were phosphorylated using T4 polynucleotide kinase (New England BioLabs). To develop high-efficient CRISPR/Cas9 knockout of the *NSUN2* gene, we designed a gRNA targeting its coding sequence. The designed gRNA sequence targeting *NSUN2* (5’– GCTGTTCGAGCACTACTACC − 3’) was subjected to BLAST sequence analysis to detect any significant similarities with other mRNAs in the reference human genome (GRCh38.p14). The designed gRNA was then inserted into the plasmid template using Golden Gate cloning. For the ligation process, 400 U of T4 DNA ligase (Thermo Fisher Scientific, Waltham, MA, USA) were used. The ligation reaction was treated with Exonuclease V (RecBCD, New England BioLabs) at 37 °C for 30 min to eliminate any remaining linearized DNA and the reaction was heat inactivated at 70 °C for 30 min. Finally, the treated ligation product was purified using the NucleoSpin^®^ Plasmid kit (Macherey-Nagel GmbH & Co. KG, Duren, Germany). Bacterial transformation with the ligated plasmids was conducted using NZY5a competent cells (NZYTech, Lisbon, Portugal) according to the manufacturer’s protocol. The successful insertion of gRNA into the plasmid vector was validated with Sanger sequencing.

### CRISPR/Cas9 genome editing

To generate *NSUN2* knockout MCF-7 cells (NSUN2*−*), CRISPR/Cas9 genome engineering was carried out in a six-well cell culture plate. The day before transfection, the cells were 80–90% confluent in a T-75 flask. A total population of 3 × 10^5^ cells were seeded 18–24 h prior to transfection in complete medium per well. The reaction included 4.0 µl Lipofectamine 3000 (Invitrogen, Thermo Fisher Scientific Inc.), Opti-MEM™ I Reduced Serum Medium (Gibco/Life Technologies), 2.5 µg of plasmid DNA and 5.0 µl of P3000 reagent. At 24 h following transfection of the construct, cells were treated with 2 µg/ml puromycin for the positive selection of the successfully modified cells. The transfected cells were harvested, and total RNA, genomic DNA (gDNA), and protein lysates were isolated for downstream analysis.

### Targeted DNA-seq

Evaluation of the *NSUN2* knockout efficiency was performed by targeted DNA sequencing using Ion Torrent™ S5 platform. For this purpose, two gene-specific primers were utilized (F: 5’– GTAGACGTCTCTTTCCTTCCTCA − 3’ and R: 5’– CTTCCTGAATCCAAACCGCTGA − 3’), designed to generate an amplicon of 312 bp, which contained the gRNA target site. Template preparation, enrichment and chip loading were carried out on an Ion Chef™ instrument, following the instructions of the manufacturer. Sequencing reads were aligned to the GRCh38 reference genome, and Integrative Genomics Viewer (IGV) was used to visualize insertions and deletions (indels) within the region of interest.

### Western blotting

Western blot was performed to assess the loss of NSUN2 protein in the KO cells (**Supplementary Data**). Cells were lysed using 1X RIPA buffer and protein concentrations in the lysates were quantified using the Bradford reagent, measuring absorbance at 595 nm. Proteins were separated by electrophoresis on 10% sodium dodecyl sulfate-polyacrylamide gels (SDS-PAGE) alongside a pre-stained protein marker to monitor migration. Following electrophoresis, proteins were transferred onto a nitrocellulose membrane, as confirmed by staining with Ponceau S solution. The membrane was blocked with a 5% non-fat milk solution to prevent non-specific binding. Subsequently, the membrane was incubated overnight at 4 °C with the primary NSUN2 Rabbit antibody mAb (44056, Cell Signaling) which has been diluted in the blocking solution according to manufacturer’s instructions. β-Actin antibody (#4967, Cell Signaling) was used as an internal loading control. After primary antibody incubation, the nitrocellulose membrane was incubated with the secondary antibody. For protein detection, the membrane was treated with an alkaline phosphatase solution containing 5-bromo-4-chloro-3-indolyl phosphate (BCIP) and nitro blue tetrazolium (NBT) substrates. Following sufficient incubation in the dark, protein bands were visualized, and results were documented.

### Nanopore direct RNA sequencing

A diverse panel of human breast cell lines was sequenced to capture the variability of m^5^C epitranscriptome in all major molecular subtypes of breast cancer (MCF-7 and BT-474 for luminal breast cancer, SK-BR-3 for HER2 + breast cancer, BT-20 and MDA-MB-231 for TNBC) as well as the non-malignant MCF-10A breast cells. For the RNA library construction, an initial amount of 500 ng mRNA sample was used from each breast cancer cell line. Sequencing libraries were prepared using the Direct RNA sequencing kit (SQK-RNA002, Oxford Nanopore Technologies Inc., ONT), following the manufacturer’s instructions. Briefly, the RT Adapter (RTA) was annealed and ligated to the 3’ poly(A) tails of mRNAs by incubating each sample at room temperature for 10 min with T4 DNA ligase (New England Biolabs Inc., Ipswich, MA, USA). Reverse transcription was then performed in a 40 µl reaction volume using a hot-lid Veriti 96-Well Fast Thermal Cycler (Applied Biosystems™). Accordingly, 1.8X Agencourt RNAClean XP beads (Beckman Coulter Inc.) were used for the purification of the synthesized cDNA sample. The RNA Adapter (RMX) was subsequently ligated to the cDNAs by incubating at room temperature for 10 min with T4 DNA ligase in a 40 µl reaction volume, followed by a second purification step with 0.4X Agencourt RNAClean XP beads. The final libraries were loaded onto a FLO-MIN106D flow cell with R9.4.1 chemistry and the nanopore sequencing was conducted on a MinION Mk1C sequencer (Oxford Nanopore Technologies Ltd, ONT). To ensure reproducibility, two sequencing runs were performed for each cell line.

### Post-processing of nanopore raw signals

To ensure consistency and avoid the introduction of potential biases during the analysis of the generated results, all sequencing datasets were processed uniformly using identical bioinformatic pipelines, including algorithm versions and parameters. Nanopore sequencing data were initially basecalled using Guppy v.6.2.1 utilizing the RNA model rna_r9.4.1_70bps_hac and applying a default read Q-score filtering threshold of 7. Only reads that passed the quality filtering were subsequently analyzed. Interactive quality assessment of the nanopore reads, including estimation of median read length, total read count, median quality score and quality scores over run time was performed with NanoPlot (De Coster and Rademakers 2023). Basecalled sequences were mapped to the reference human transcriptome (NCBI RefSeq assembly: GCF_000001405.40) using minimap2 v. 2.22 (Li 2018) and IsoQuant (Prjibelski et al. 2023) was employed for expression analysis and transcript quantification. Indexing and alignment of nanopore events to reference k-mers was carried out using nanopolish v. 0.14.

### m^5^C stoichiometry analysis and mRNA expression

The aligned nanopore reads were analyzed using CHEUI, a two-stage neural network software designed to detect and quantify m^5^C sites in nanopore RNA sequencing datasets (Acera Mateos et al. 2024). The minimum coverage of eight sequencing reads was applied for any site to be considered as m^5^C. Differential methylation levels between breast cancer cell lines and the non-cancerous MCF-10 A cell line were assessed using a Wilcoxon rank-sum test facilitated by CHEUI-diff module, using the default parameters. Each identified m^5^C site was assigned a prediction probability score ranging from 0 to 1. It is important to note that all detected m^5^C sites demonstrated consistent stoichiometry and modification probability across replicates. It should be mentioned that a probability cutoff of 0.9999 for CHEUI-solo Model 2 corresponds to a false discovery rate (FDR) equal to 0.000384 for m^5^C, as determined by a permutation test (Acera Mateos et al. 2024). Hence, among the detected sites in our study, only those exhibiting prediction probability > 0.9999 were considered highly confident m^5^C. Exploiting the ability of CHEUI to provide the 9-mer (m^5^C-centered) of each detected m^5^C, besides the overall m^5^C epitranscriptomic analysis, we also implemented motif-specific analysis of m^5^C sites in both WT and KO conditions.

To investigate the global impact of m^5^C methylation on mRNA abundance, we analyzed the correlation between differential methylation levels (WT vs. KO) and differential expression of transcripts. Transcript abundance was assessed with the Transcripts Per Million (TPM) method. Then, methylation stoichiometry was stratified into five intervals (0.0–0.2, 0.2–0.4, 0.4–0.6, 0.6–0.8, and 0.8–1.0), representing incremental methylation levels. TPM distributions across these stoichiometric ranges were visualized using split-violin plots to assess the relationship between m^5^C methylation intensity and mRNA expression.

### Differential methylation, co-methylation and functional enrichment analysis

Differential methylation of mRNA m^5^C sites between the non-cancerous breast cell line (MCF-10 A) and various breast cancer cell lines (SK-BR-3, MCF-7, MDA-MB-231, BT-474, BT-20) was assessed using the CHEUI algorithm (Acera Mateos et al. 2024). Specifically, we determined the absolute number of raw sequencing reads associated with differentially modified m^5^C sites based on CHEUI m^5^C stoichiometry and coverage and calculated the log2-transformed fold change (log2FC) of m^5^C methylation for each site between non-cancerous and cancerous conditions. Sites with significant hypo- or hypermethylation were identified using cutoffs of|log2FC|≥1.0 and an adjusted *p*-value of *p* < 0.001. Differential methylation of m^5^C sites was evaluated for each cancerous cell line compared to non-cancerous breast cells MCF-10A using volcano plots generated in R with the ggplot2 package. Next, we estimated the concordance of m^5^C mRNA methylation between breast cancer cell lines by calculating Pearson’s correlation coefficient and constructing a pairwise scatterplot using ggplot2. The top 20 significant hypo- and the top 20 hypermethylated m^5^C sites from each breast cancer cell line were extracted from the correlation analysis. Genes corresponding to all significantly hypo- and hypermethylated m^5^C sites were then used for functional analysis of human genes with significantly modified m^5^C sites in breast cancer, utilizing ShinyGO v.0.80 with default settings (0.05 FDR-adjusted *p*-value as threshold) (Ge et al. 2020).

### Expression analysis of NSUN2 targets

To evaluate the expression of NSUN2 target genes (*FBL*,* DNMT2*,* YBX1*,* TRMT112*,* TP53*,* ALYREF*,* IGF2BP1*,* LIN28A*,* NOP2*) in WT and KO cells, SYBR Green-based quantitative PCR (qPCR) assays were performed using gene-specific primers (Supplementary Table S1). Total RNA (5 µg) was extracted from WT and KO cells and used as template for reverse transcription (RT). A 12 µl reaction mixture was prepared, consisting of 10 µl total RNA, 1 µl oligo-dT (10 µM) and 1 µl of dNTP mix (10 mM each). The mixture was incubated at 65 °C for 5 min in a Veriti™ 96-Well Fast Thermal Cycler (Applied Biosystems™), followed by cooling on ice for 2 min. The final reaction volume was adjusted to 20 µl by adding 2 µl of nuclease-free H2O, 4 µl of 5X First-Strand Buffer, 1 µl of DTT (100 mM), 10U of RNaseOUT inhibitor (Invitrogen™, Thermo Fisher Scientific Inc.) and 100U of SuperScript™ III (Invitrogen™, Thermo Fisher Scientific Inc.). RT was performed at 50 °C for 60 min, followed by enzyme inactivation at 70 °C for 15 min. The generated cDNA was diluted 1:10 in nuclease-free H2O and was used as the template for qPCR. qPCR was conducted using the QuantStudio™ 5 Real-Time PCR System (Applied Biosystems™) in 10 µl reaction volumes, containing 5 µl 2X Kapa SYBR^®^ Fast qPCR Master Mix (Kapa Biosystems, Inc., Woburn, MA, USA), 2 µM of each primer, 2 µl of nuclease-free H2O as well as 1 µl of diluted cDNA template. The thermal cycling protocol included an initial denaturation at 95 °C for 3 min, followed by 40 cycles of 95 °C for 3 s and 60 °C for 30 s. Fold changes (FC) in target gene expression between two conditions were assessed using the 2^−ΔΔCt^ method, with human *GAPDH* mRNA serving as the internal reference control gene. Melt-curve analysis confirmed primer specificity.

### mRNA stability assay

Both WT and KO cells were plated in 24-well plates and treated with actinomycin D (Gibco™, Thermo Fisher Scientific Inc.) at a concentration of 5 µg/ml for 0, 3, 6, and 9 h to inhibit transcription. After treatment, cells were collected, and total RNA was isolated. RNA was reverse transcribed using SuperScript™ III Reverse Transcriptase in 20 µl reaction volumes as described previously. First-strand cDNA was used as a template for qPCR with gene-specific primers targeting mRNAs containing m^5^C sites (Supplementary Table S2). *GAPDH* was used as internal reference control to normalize expression obtained by qPCR. Relative expression for mRNA targets was calculated using the 2^−ΔΔCt^ method, and results were expressed in relative quantification (RQ) units. Finally, the proportion of remaining mRNA in each time point relative to time 0 was calculated.

## Results

### Sequencing of native full-length mRNAs provided the m^5^C methylome of breast cancer subtypes

**Fig. 1:**
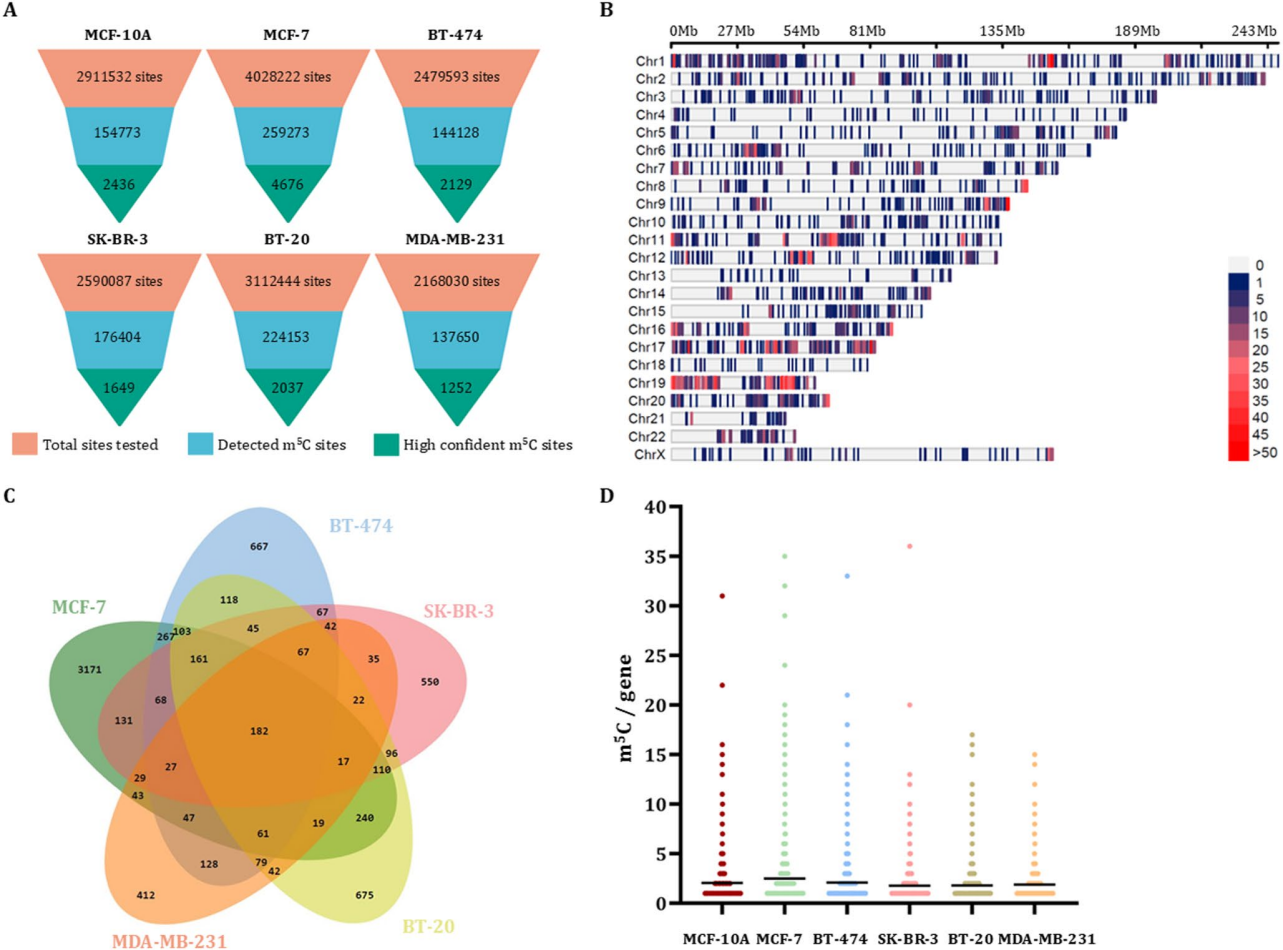
Descriptive analysis of the nanopore sequencing data generated from the diverse panel of breast cell lines representing all major molecular subtypes of breast cancer (MCF-7 and BT-474 for luminal breast cancer, SK-BR-3 for HER2 + breast cancer, BT-20 and MDA-MB-231 for TNBC) as well as the non-malignant MCF-10 A breast cells. **(A)** Funnel plot of the m^5^C sites that were investigated in the breast cell lines. Light red color is used to present the total number of cytosine sites that have been tested for m^5^C methylation with CHEUI in each cell line. Light blue corresponds to the percentage of the identified m^5^C sites, while green is used for the highly confident sites (probability > 0.9999). **(B)** Genomic distribution of the highly confident (probability > 0.9999) m^5^C sites that reside within human mRNAs. The horizontal axis represents the chromosome length in Mb. Different colors correspond to the number of m^5^C methylation within a 27 Mb window size. **(C)** Venn diagram representing the number of unique and common highly confident (probability > 0.9999) m^5^C-methylated genes among the five breast cancer cell lines. **(D)** Demonstration of the number of m^5^C sites per gene. Black horizontal lines show the median value of m^5^C sites per gene in each cell line

In the present study, we sequenced six distinct RNA libraries, derived from five cancerous human cell lines, each representing a different molecular subtype of breast cancer (BT-20, MDA-MB-231, BT-474, MCF-7 and SK-BR-3) and one from the non-cancerous breast cell line MCF-10 A. The amount of raw sequencing data produced by nanopore varied across the different libraries, ranging from 750.00 Mb to 1.3 Gb. Transcriptome-wide m^5^C sites were detected utilizing the ionic current signals produced by nanopore sequencing, which enable the identification of m^5^C sites both at transcriptome-wide and transcript-specific level. Analysis of > 2,000,000 cytosines, using a minimum coverage of 8 reads in each cell line, revealed methylation at < 10% of tested sites (Fig. 1A). To mitigate false positives arising from nanopore current signal artifacts, stringent filtering was implemented. Only sites with a per-site probability > 0.9999 (FDR < 0.01%; Supplementary Table S3) were retained as high-confidence m^5^C sites for downstream analysis. This probability threshold was selected based on the empirical validation described by Mateos et al., who demonstrated that CHEUI predictions with a probability score above 0.9999 exhibit near-zero FDR in benchmark datasets. By adopting this cutoff, we prioritized specificity over sensitivity to ensure that the detected m⁵C sites reflect true positive signals. This criterion classified approximately 1% of initially detected sites as highly confident m^5^C (Fig. 1A). Although this approach ensures robustness, it may exclude low-confidence or transiently modified sites, necessitating complementary methods for comprehensive epitranscriptomic profiling.

### Quantitative profiling and m^5^C distribution of mRNAs in breast cancer

Our analysis revealed that both density and distribution of m^5^C sites vary among mRNAs. mRNAs transcribed from genes located at chromosomes 1, 11, 17 and 19 exhibited an increased number of m^5^C sites (Fig. 1B). Additionally, comparative analysis revealed that only 182 highly confident m^5^C sites within mRNAs were common among the investigated breast cancer cell lines (Fig. 1C). However, it should be noted that the number of unique or shared identified m^5^C sites between cell lines may be affected by the stringent probability cutoff that was applied (> 0.9999). Interestingly, across all the analyzed breast cells, a median of two m^5^C sites per gene (aggregating all splice variants) was observed. Notably, MCF-7 deviated from the observed trend, displaying a higher median of three m^5^C sites per gene compared to the two-site median in other cancerous breast cell lines (Fig. 1D).

**Fig. 2:**
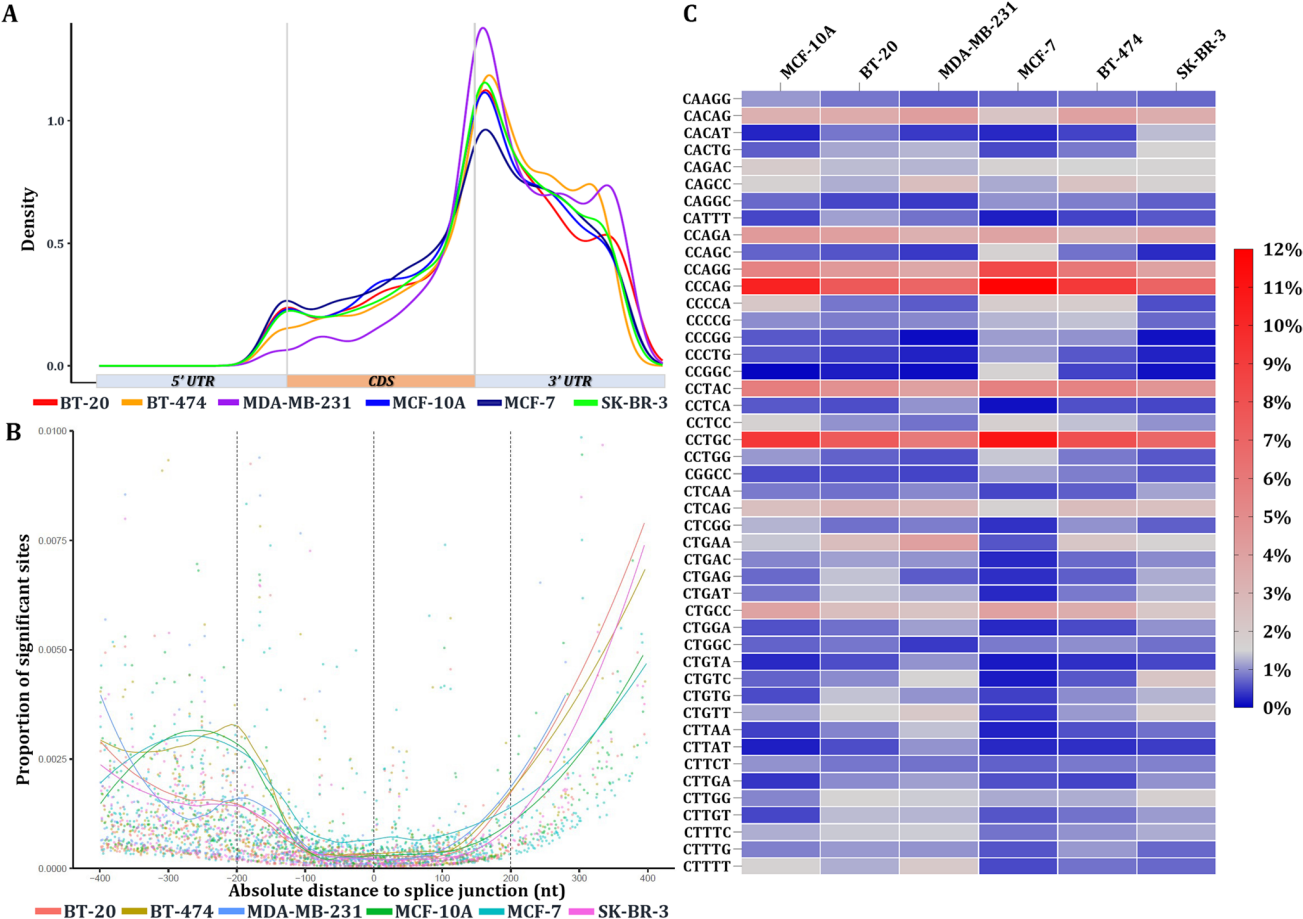
Distribution of the highly confident (probability > 0.9999) m^5^C sites on human mRNAs and presentation of the m^5^C-related motifs. **(A)** Metaplot illustrating the relative abundance of m^5^C sites within the individual regions of mRNA transcripts (UTR: untranslated region, CDS: protein-coding region). **(B)** Correlation between the detected m^5^C sites and their distance from mRNA splice junctions. **(C)** Heatmap showing the relative abundance (%) of each motif harboring highly confident m^5^C sites. The m^5^C nucleotide is at the beginning of each presented motif

The spatial distribution of m^5^C on mRNAs across all the breast cell lines revealed consistent mRNA region patterns. Specifically, m^5^C are located across the whole sequence of mRNAs, being predominantly enriched in the 3’ untranslated region (3’ UTR) and especially near the stop codon (Fig. 2A). Notably, positional analysis revealed enrichment of m^5^C sites in regions flanking splice junction sites, extending from 100 to 400 nucleotides both upstream and downstream from the splice junction sites. On the contrary, minimal proportion of highly confident m^5^C sites was observed within a window of ± 100 nt from splice junctions (Fig. 2B**).** This distribution pattern of m^5^C deposits may indicate a potential regulatory role in modulating pre-mRNA splicing or stabilizing specific mRNA transcript variants.

### m^5^C modifications reside within NSUN2-independent recognition motifs

To further investigate the association of m^5^C sites with consensus sequences, we systematically assessed the abundance of distinct 5-mers containing the m^5^C site positioned at the beginning of the motif. We observed that most of these motifs were consistently methylated across the different cell lines, however, several of them exhibited significantly higher m^5^C levels in specific cell types, suggesting a bimodal deposition influenced by both motif idiosyncrasy and cell type (Fig. 2C).

Motif sequence analysis unveiled that T-rich motifs or the ones harboring m^5^C followed by G in the + 1 position were rarely observed. In contrast, 5′-***C***CNNN-3′ motifs (N = any base) displayed variable m^5^C methylation levels across cell lines, with 5′-***C***CAGC-3′, 5′-***C***CCAG-3′, and 5′-***C***CTGC-3′ emerging as the predominant 5-mers containing high-confidence m^5^C sites. These conserved motifs—consistent across cell lines—suggest preferential targeting by m^5^C methyltransferases, underscoring sequence context as a critical determinant of methylation specificity (Fig. 2C).

### Investigation of the m^5^C motif-dependencies in NSUN2- breast cancer cells

**Fig. 3:**
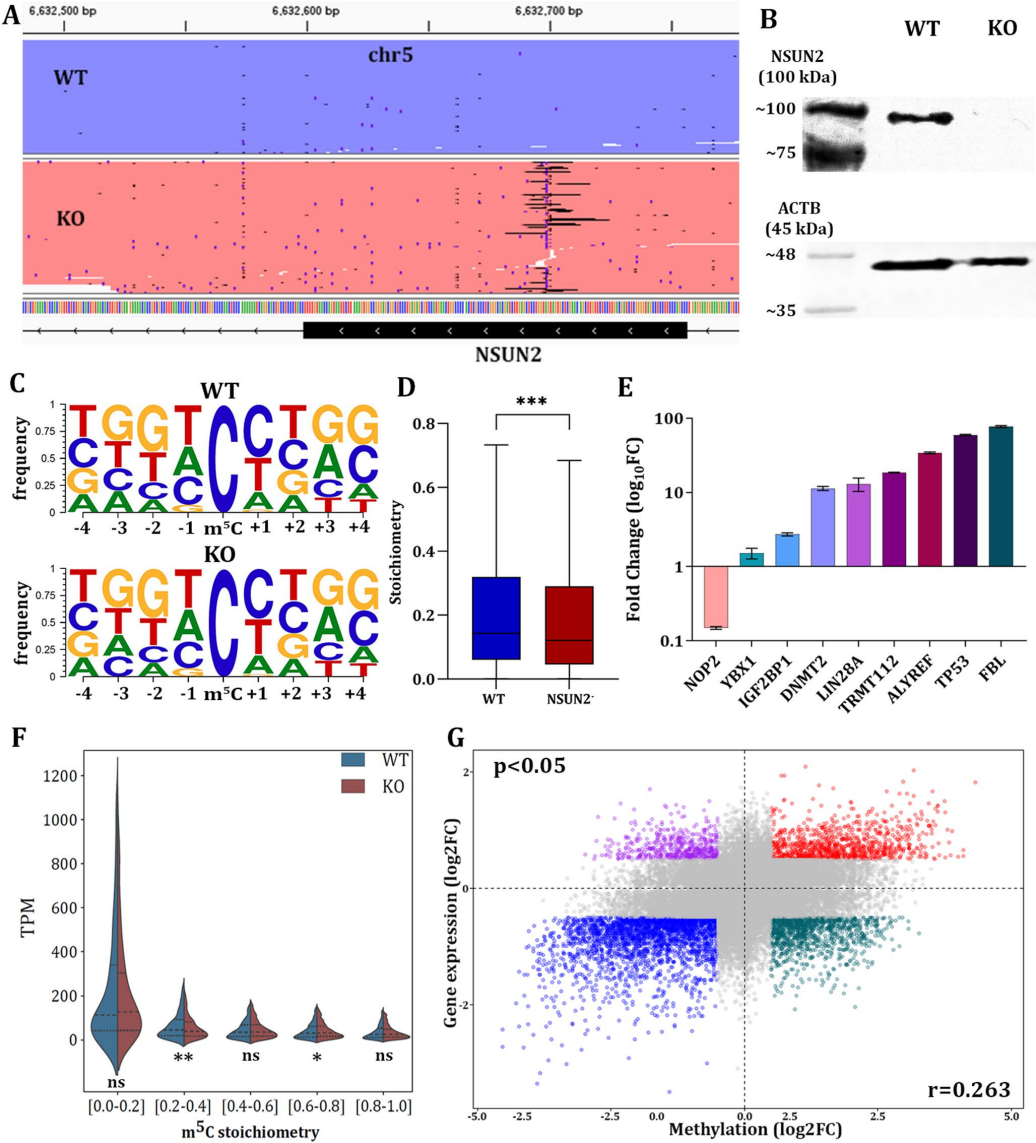
Assessment of NSUN2^―^ knockout, evaluation of its motif-dependencies, investigation of its canonical target expression and m^5^C methylation status analysis. **(A)** IGV visualization of aligned NGS reads from WT and KO breast cells, demonstrating the efficiency of the knockout. The genomic region that is shown is equal to 312 bp, which is the amplicon size that was sequenced. For visual purposes, aligned reads from WT and KO cells are shown in blue and red color, respectively. **(B)** Western blot results for NSUN2 and ACTB expression in both WT and KO cells. **(C)** Motifplots for both WT and KO cells presenting all the 9mer-based motifs detected by CHEUI, centered at the highly confident m^5^C site. The sequence content is displayed as a pictogram. For each cell line the letter height is proportional to the frequency of the corresponding nucleotide at the given position. For each position bases are listed in descending order of frequency from top to bottom. **(D)** Boxplot depicting the differences in m^5^C stoichiometries of the highly confident sites between the WT and NSUN2- cells. *P*-value is shown with three asterisks (***) and corresponds to the significance level. (*p*-value < 0.001). **(E)** Graphical representation of the fold change values corresponding to the relative expression of the canonical NSUN2 targets in WT cells as compared to KO cells. Relative gene expression was assessed by qPCR using the 2^−ΔΔCt^ method and *GAPDH* as housekeeping gene. qPCR experiments and downstream analysis were carried out using three technical replicates. **(F)** Half-violin plots showing the association of TPM values and m^5^C stoichiometry for both WT and KO conditions. Mann-Whitney U test was performed to assess the significance of TPM differences between WT and KO in each stoichiometry group. The symbol “*” is used to highlight a significance level of *p*-value < 0.05, “**” corresponds to a significance level of *p*-value < 0.01, while “ns” is used to denote non-significant differences. **(G)** Association of m^5^C methylation levels with overall gene expression levels. Red indicates hypermethylated sites within overexpressed transcripts, while blue represents hypomethylated sites within underexpressed transcripts. Purple highlights hypomethylated sites within overexpressed transcripts, and green corresponds to hypermethylated sites within underexpressed transcripts

The biological role of NSUN2 as a major m^5^C writer and its motif dependencies in mRNAs were investigated using both WT and NSUN2- cells. The efficiency of *NSUN2* depletion was evaluated with targeted DNA-seq using as template gDNA from both conditions (Fig. 3A). Additionally, western blotting confirmed the loss of NSUN2 protein on KO cells (Fig. 3B). Clustering analysis of the m^5^C-centered 9-mers provided by CHEUI in both WT and KO cells unveiled an intriguing preference for thymidine or adenosine at the − 1 position and guanosine or thymidine at -2. At the same time, no specific nucleotide preference was observed at positions − 3 and − 4 from each m^5^C site (Fig. 3C). Conversely, positions + 1 and + 2 exhibited a preference for cytosine or thymidine, with a prevailing G-rich region at positions + 3 and/or + 4 (Fig. 3C). Importantly, no differences in 9-mer motifs were observed in NSUN2- cells. Furthermore, the attenuated m^5^C stoichiometries of mRNAs in NSUN2- cells, compared to the respective methylation levels in WT cells, points towards the direction that NSUN2 serves as a major and conserved m^5^C writer in human cells. Interestingly, motif analysis of the sequencing datasets unveiled that 5’-***C***NGGG-3’ motif, which represents the predominant NSUN2 target, demonstrates significant hypomethylation (*P* < 0.001), between the WT and the KO cells (Fig. 3D).

### Regulatory effects of NSUN2 on its canonical mRNA targets

To assess the impact of NSUN2 knockout, qPCR was performed on nine well-established NSUN2 downstream targets and relative gene expression was calculated using the 2^-ΔΔCt^ method. Amplification findings revealed significant differences in expression between WT and KO cells (Fig. 3E). Specifically, most of the investigated genes exhibited higher expression in WT cells, whereas only the *NOP2* gene showed notably reduced expression in WT cells. The *YBX1* m^5^C reader displayed a slight overexpression in WT cells, while *IGF2BP1* was expressed at approximately twice the level in WT compared to KO cells (Fig. 3E). Similarly, *DNMT2*, *LIN28A*, and *TRMT112* were significantly overexpressed in WT cells. Notably, three additional targets *— ALYREF*, *TP53* and *FBL* — demonstrated more than 50-fold higher expression in WT cells. These findings are indicative of the great impact of NSUN2 for the regulation of several genes. The substantial reduction of mRNA levels upon *NSUN2* knockout indicates that NSUN2-mediated RNA methylation may play a pivotal role in maintaining proper gene expression networks, particularly in pathways related to RNA processing and translation.

### m^5^C methylation exhibits a weak positive correlation with mRNA abundance

To characterize m^5^C methylation patterns in relation to transcript abundance, we performed a descriptive analysis of mRNA expression (transcript-per-million values, TPM) across stratified m^5^C stoichiometry subgroups (0.0–0.2, 0.2–0.4, 0.4–0.6, 0.6–0.8 and 0.8–1.0) in WT and KO cells (Fig. 3F). While mRNAs in the lowest methylation subgroup (0.0–0.2) exhibited elevated median TPM values relative to higher stoichiometry subgroups, transcript abundance showed no progressive trend across increasing methylation levels. To directly evaluate the regulatory impact of m^5^C methylation, we calculated the log2 fold changes (log2FC) in both m^5^C stoichiometry and transcript expression between WT and NSUN2- conditions. Correlation analysis revealed a weak but statistically significant positive association (Pearson correlation coefficient, *r* = 0.263, *p* < 0.05) between differential m^5^C methylation and changes in transcript abundance (Fig. 3G). These observations suggest that, at a global level, m^5^C methylation does not robustly correlate with steady-state mRNA abundance under the tested conditions, though further mechanistic studies are warranted to resolve context- or transcript-specific regulatory roles.

### NSUN2 influences mRNA stability in a transcript-specific manner

**Fig. 4:**
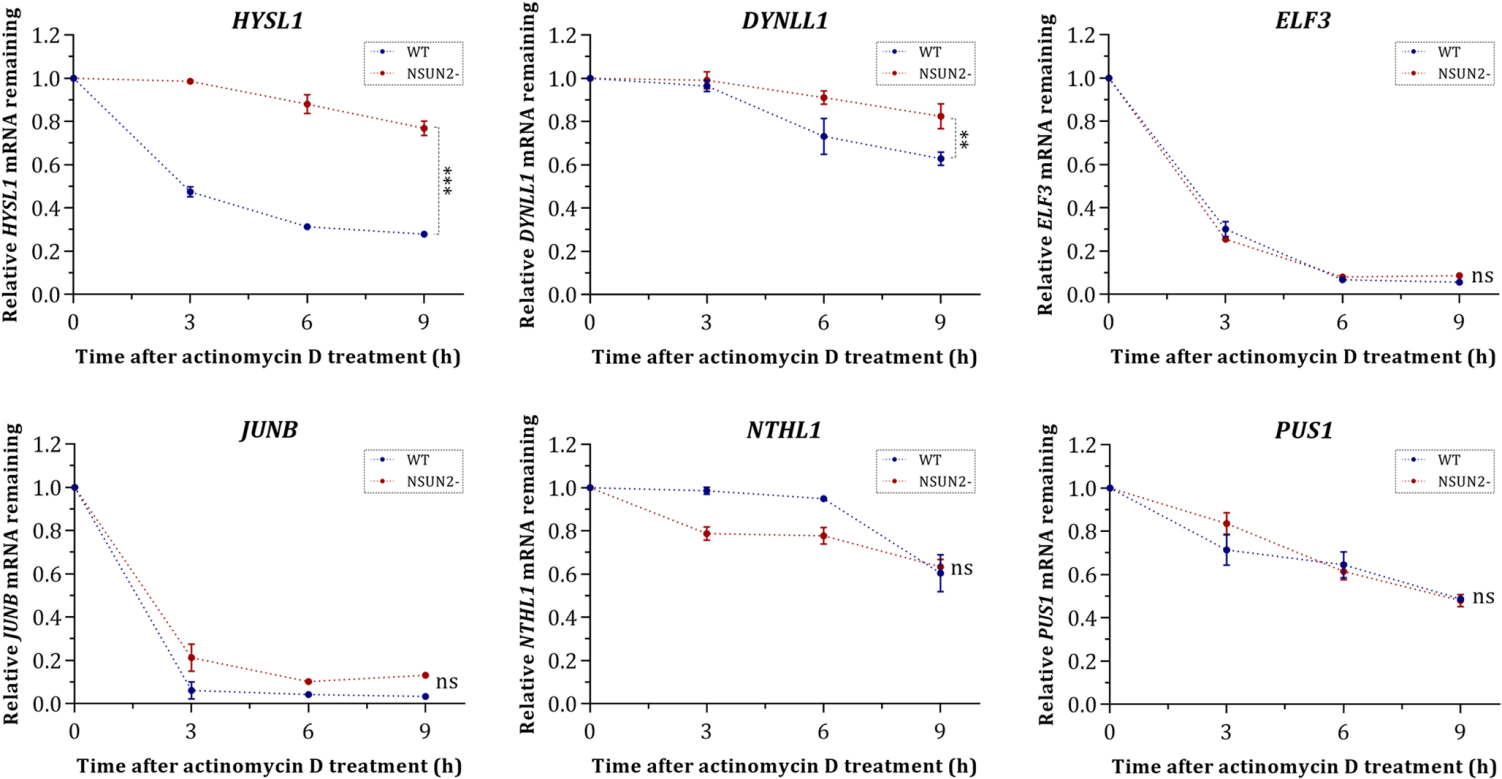
Graphical representation of NSUN2 loss effect on mRNA stability after treatment of WT and KO cells with actinomycin D. Data was normalized using the 2^−ΔΔCt^ method and *GAPDH* as internal reference gene. Remaining mRNA levels following actinomycin D treatment were calculated relative to timepoint 0 across the indicated timepoints, with error bars representing standard deviation from three biological replicates. The symbol “*” is used to highlight a significance level of *p*-value < 0.05, whereas “ns” is used for non-significant differences between the two investigated conditions

To investigate the role of NSUN2-mediated m^5^C methylation in mRNA stability, we performed actinomycin D-based mRNA decay assays. Our results revealed a transcript-specific effect of NSUN2 depletion on mRNA stability. *ELF3* and *JUNB* mRNAs exhibited no significant difference in degradation rates between WT and KO cells (Fig. 4). However, both transcripts displayed rapid degradation in both conditions, suggesting that their stability is independent of NSUN2-mediated methylation. Notably, *NTHL1* and *PUS1* demonstrated a slight stabilization in WT cells than in KO cells, as indicated by their higher relative mRNA abundance over time. However, statistical analysis revealed no significant differentiation between WT and NSUN2- cells both in *NTHL1* and *PUS1*. Conversely, *DYNLL1* and *HYSL1* displayed increased stability in NSUN2 KO cells, as indicated by a reduced slope in linear regression analysis, suggesting that m^5^C deposition by NSUN2 promotes their degradation (Fig. 4). These findings highlight a context-dependent role for NSUN2 in mRNA turnover, where m^5^C modifications can either stabilize or destabilize specific transcripts.

### Breast cancer subtypes display aberrant m^5^C methylation patterns

**Fig. 5:**
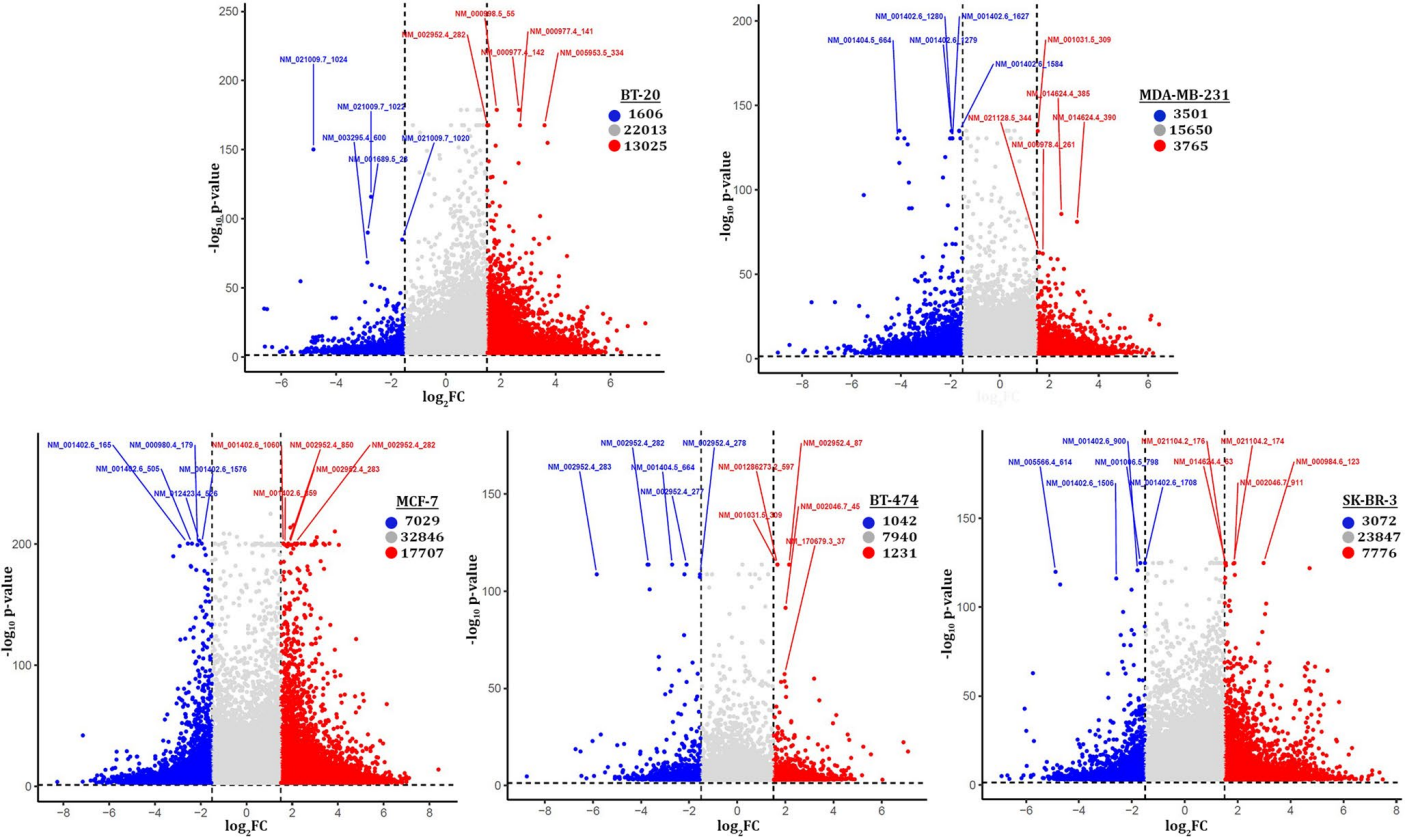
Schematic demonstration of the differentially methylated m^5^C sites between each cancerous cell line and the non-cancerous MCF-10 A cells. The GenBank^®^ transcript ID and transcriptomic coordinates of the 5 most significant hyper- and hypomethylated m^5^C sites are also shown for each breast cancer cell line

Differential m^5^C methylation analysis between breast cancer cell lines (SK-BR-3, MCF-7, MDA-MB-231, BT-474, BT-20) and the non-cancerous MCF-10 A cells revealed numerous differentially methylated m^5^C sites. An overall m^5^C hypermethylation was the predominant trend in all breast cancer cell lines (Fig. 5). The most significant hypermethylation was observed in MCF-7 (17707 sites) and BT-20 (13025 sites) cells. Concurrently, BT-20 cells exhibited the second lowest number of hypomethylated sites (1606), indicating an overall hypermethylation of mRNAs in BT-20 cells. The least variation in m^5^C methylation was observed in MDA-MB-231 cells with 3765 hypermethylated and 3501 hypomethylated sites, and in BT-474 cells with 1231 hypermethylated and 1041 hypomethylated sites (Fig. 5). Interestingly, BT-474 cells displayed the lowest number of significantly methylated m^5^C sites. In SK-BR-3, a total of 10848 m^5^C sites were found to be aberrantly methylated, with 7776 showing significantly increased methylation and 3072 showing significant hypomethylation (Fig. 5).

**Fig. 6:**
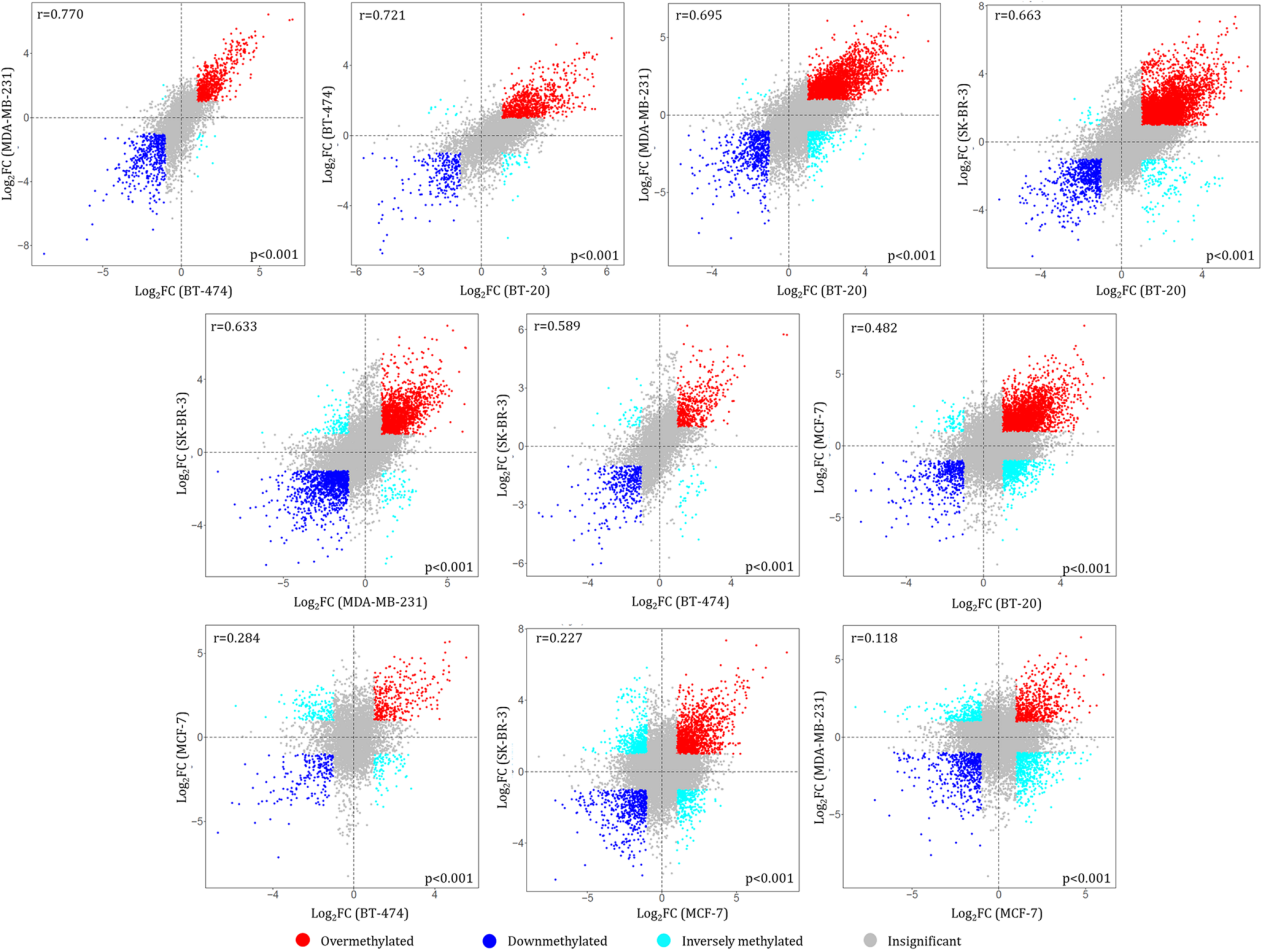
Pairwise scatterplot matrix showing differential methylation of transcriptomic sites in breast cancer cell lines according to log2-fold change (Log2FC) values relative to non-cancerous MCF-10 A cells. Positive correlations between the investigated cell lines are shown in red, negative correlations in blue, whereas the inversely methylated m^5^C points are colored in cyan. The correlation was calculated by the Pearson coefficient with a *p*-value < 0.01

To evaluate the concordance in m^5^C methylation patterns across all possible pairs of breast cancer cell lines, we constructed a pairwise scatterplot matrix using log-transformed differential m^5^C methylation data by direct comparison between the non-cancerous breast cell line and each corresponding breast cancer cell line (Fig. 6). Overall, we observed low to moderate positive correlations among differentially methylated m^5^C sites between most of the breast cancer cell lines, as indicated by Pearson correlation coefficients ranging from *r* = 0.118 to *r* = 0.770 (Fig. 6). Specifically, we noted a slight or negligible positive linear relationship of the differentially methylated m^5^C sites between MCF-7 and BT-474 (*r* = 0.284), MDA-MB-231 and MCF-7 (*r* = 0.118), as well as SK-BR-3 and MCF-7 (*r* = 0.227). However, a moderate correlation was found between MCF-7 and BT-20 (*r* = 0.482). These comparisons indicate that the m^5^C methylation pattern of MCF-7 mRNAs is notably distinct from those of other breast cancer cell lines, except for BT-20 cells. Furthermore, the strongest correlation of differentially methylated m^5^C points was detected between cancer cell lines MDA-MB-231 and BT-474 (*r* = 0.770), BT-474 and BT-20 (*r* = 0.721), as well as MDA-MB-231 and BT-20 (*r* = 0.695) (Fig. 6).

### Subtype-specific m^5^C methylation reveals context-dependent regulation yet implicates conserved functional pathways

**Fig. 7:**
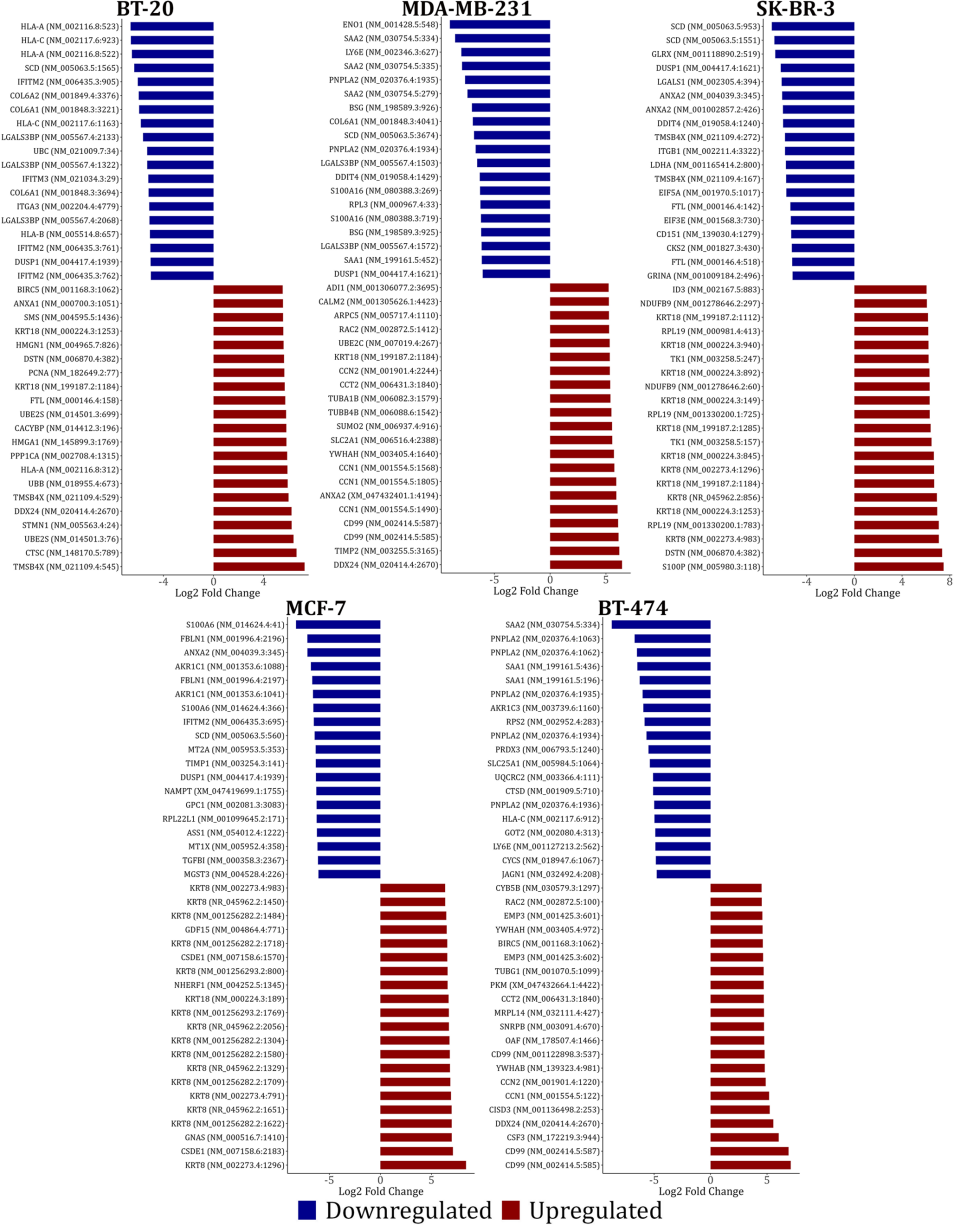
Reverse barplots highlighting the 20 most hypo- and hypermethylated m^5^C sites that were detected in each breast cancer molecular subtype relative to the non-cancerous MCF-10 A cell line. Blue and red bars represent hypo- and hypermethylated m^5^C transcriptomic sites, respectively

To decipher m^5^C methylation landscapes across breast cancer subtypes, we identified hyper- and hypomethylated mRNA sites across 5 molecularly distinct breast cancer cells lines. Bidirectional horizontal bar plots illustrate the top 20 significantly hyper- and hypomethylated transcripts in each cell line, revealing a minimal overlap between subtypes (< 1% shared transcripts), consistent with cell type-specific regulation of m^5^C deposition (Fig. 7). To further evaluate the functional relevance of these methylation patterns, we performed both Gene Ontology (GO) and Kyoto Encyclopedia of Genes and Genomes (KEGG) pathway enrichment analysis of genes harboring subtype-specific hyper- or hypomethylated m^5^C sites. Despite the lack of overlap in individual methylated transcripts, consensus biological processes emerged across subtypes, including translation regulation, ribosomal assembly, and RNA binding (**Supplementary Data**). Interestingly, KEGG pathway analysis revealed that m^5^C methylation may be involved in the metabolic reprogramming of breast cancer cells, as well as in the regulation of key oncogenic pathways such as the ErbB, apoptosis, and DNA repair mechanisms (**Supplementary Data**).

## Discussion

Epitranscriptomics, a rapidly evolving branch of omics, explores how chemical modifications dynamically regulate and control RNA function. These modifications typically involve the methylation of distinct RNA nucleotides—such as m⁶A and m⁵C—and are orchestrated by specific enzymes and effector proteins that add, remove, and interpret these marks, forming a precisely regulated cellular procedure (Boo and Kim 2020). Recent studies have shown that epitranscriptomic modifications are highly dynamic, responding to environmental stimuli and developmental cues. For example, the most well-studied RNA modification, m⁶A, has been found to be implicated in mRNA splicing, export, and decay, while modifications on non-coding RNAs facilitate in maintenance of structural integrity and ensure accurate protein synthesis. More importantly, dysregulation of RNA modifications has been linked to various diseases, including cancer, metabolic disorders, and neurological conditions (Gao and Fang 2021; Wilkinson et al. 2022). Recent studies have highlighted the role of m^5^C in gene expression, cell proliferation, and metabolism, linking it to various malignancies, including gynecologic cancers (Gu et al. 2023). Most of these studies are mainly focused on elucidating the proteins involved in the m^5^C methylation machinery, such as the NSUN2 methyltransferase, and their impact on mRNA methylation. Despite ongoing efforts, accurate profiling of m⁵C in mRNA remains challenging because current detection methods cannot reliably distinguish them from other RNA modifications, lacking the required sensitivity and specificity for precise mapping (Trixl and Lusser 2019). Advancements in high-throughput sequencing and computational algorithms are critical to unravel the mechanistic roles of the epitranscriptome in various biological processes and diseases (Wu et al. 2024). This study presents the first comprehensive transcriptome-wide mapping of the m^5^C epitranscriptome in breast cancer, by applying nanopore sequencing to established cell line models representing all major molecular subtypes. This approach achieves single-nucleotide resolution mapping and absolute quantification of m^5^C stoichiometry across the transcriptome (Wu et al. 2024).

Previous studies have reported varying numbers of m^5^C sites from hundreds to thousands (Hartstock et al. 2023). Using NGS methodologies like miCLIP-seq or bisulfite sequencing, we can identify up to 95000 m^5^C sites on human mRNAs (Huang et al. 2021). In our study, we employed nanopore direct RNA sequencing, successfully identifying over 100000 m^5^C sites per cell line; however, only 1% of these were classified as high-confidence sites (probability > 0.9999). This finding aligns with MePMe-seq data, which identified 1276 high-confidence m^5^C sites in HeLa cells. This agreement validates the robustness and precision of our methodology, further supported by reproducibility across different experimental platforms (Hartstock et al. 2023). Our analysis identified significant enrichment of m^5^C modifications on mRNAs encoded by genes located on chromosomes 11, 17, and 19. This spatial clustering suggests a coordinated interplay between genetic, epigenetic, and epitranscriptomic mechanisms, potentially fine-tuning oncogenic transcript stability or translation in cancer. Notably, mRNA transcript variants of genes reside on chromosome 19 displayed also increased m^5^C methylation in colorectal cancer HCT-15 cells, indicating conserved targeting of these loci across multiple cancer types (Lin et al. 2023). This notion is unsurprising as these chromosomes contain multiple cancer-related gene loci, including several genes and regulatory elements that are crucial for cancer development.

Defining the sequence motifs targeted by m^5^C regulatory proteins is crucial for understanding the *modus operandi* of the m^5^C methylation process. It is well-documented that the NSUN family members are responsible for m^5^C incorporation into mRNAs (Cui et al. 2024; Guarnacci et al. 2024). Type I m^5^C sites, dependent on NSUN2, are characterized by a 3’ G-rich triplet (3’ NGGG) motif while Type II sites, dependent on NSUN6, are described by the 3’ TCCA motif (Huang et al. 2019). Using CRISPR/Cas9 to knock out the *NSUN2* gene, we explored the m^5^C consensus sequences in the human transcriptome. Bioinformatics analysis of the knockout datasets revealed decreased stoichiometry of Type I m^5^C sites, confirming that NSUN2 demonstrates a key m^5^C writer in breast cancer cells. This observation is consistent with findings from colorectal cancer studies (Lin et al. 2023). Notably, in our study the 5’-***C***CCAG-3’ motif emerged as the most abundant 5-mer in all breast cell lines, indicating that additional m^5^C writers participate in methylation of mRNAs. This hypothesis is supported by studies employing different methodologies, such as bs-RNA, directly linking NSUN1 and NSUN5 with m^5^C methylation of mRNAs (Liu et al. 2023). Additional m^5^C motifs have also been reported, with Type III and IV sites linked to NSUN5 and NSUN1, characterized by the 5’-UNGCCANNUG-3’ and 5’-YYCGANKU-3’ (where Y = U or C and K = G or U) motifs, respectively (Guarnacci and Preiss 2024).

A key finding of the current study is the determination of m^5^C distribution across mRNA regions in the investigated breast cancer subtypes. Enrichment of m^5^C sites within the 3’ UTR and CDS suggest that methylation may modulate key regulatory mechanisms, such as mRNA stability and translation efficiency, adding a potential novel layer of regulation to cellular functions (Zheng et al. 2023). Recent findings have highlighted that m^5^C deposition can either inhibit or promote mRNA decay by influencing RNA structure, ribonucleoprotein interactions, or RNA degradation pathways (Chen et al. 2024; Mei et al. 2020; Wang et al. 2023; Zhang et al. 2012, 2023). One possible mechanism for this stabilization is the recruitment of m^5^C readers, such as YBX1, which we found to be upregulated in WT cells compared to KO cells. YBX1 has been shown to bind m^5^C-modified transcripts and enhance their stability, which may explain why certain NSUN2-dependent mRNAs exhibit reduced degradation rates in WT cells. This aligns with previous reports in which YBX1-mediated mRNA stabilization contributes to tumor progression by promoting oncogenic pathways (Zhang et al. 2024). Contrary to the established role of NSUN2 in stabilizing mRNAs via m^5^C deposition, our findings reveal that m^5^C can paradoxically act as a destabilizing signal for specific transcripts. In our study, NSUN2 depletion increased the stability of *DYNLL1* and *HYSL1* mRNAs, indicating that methylation at these loci recruits promotes mRNA decay or disrupts protective RNA-protein interactions (Fig. 4). This potential destabilizing role of NSUN2-mediated m^5^C methylation has been documented in a prior study, where m^5^C modification of *IRF3* mRNA promoted its degradation through ALYREF-dependent nuclear export (Wang et al. 2023). These findings underscore the multifaceted role of NSUN2-mediated m^5^C methylation, which variably stabilizes or destabilizes mRNAs depending on transcript identity, interacting effector proteins and compensatory regulatory pathways. This mechanistic plasticity challenges the notion of m^5^C as a universal stabilizing signal, establishing it instead as a dynamic modulator of mRNA fate shaped by competing epitranscriptomic networks.

Although previous studies have predominantly focused on the differential expression levels of m^5^C writers to evaluate methylation patterns in human cancers, our study primarily investigates the methylation status of mRNAs. Differential methylation analysis of the m^5^C sites between breast cancer cell lines and non-cancerous MCF-10 A cells showed that most differentially methylated sites were hypermethylated in cancer cells. Our results indicate a significant concordance of differentially methylated m^5^C sites between most of the breast cancer cell lines, with Pearson correlation coefficients ranging from 0.4 to 0.7. However, MCF-7 cells exhibited a distinct methylation profile, showing moderate correlation only with BT-20 cells (*r* = 0.482). This similarity may reflect conserved oncogenic pathways, which activate common epitranscriptomic programs across different in terms of molecular characteristics and clinical outcome subtypes. Collectively these data indicate that common m^5^C epitranscriptomic patterns are preserved, however they minimally affect global gene expression as illustrated by the comparison of differential methylation and expression levels in NSUN2 WT and KO cells. Although our study identified minimal overlap in common hyper- or hypomethylated m^5^C sites across breast cancer subtypes, functional enrichment analysis uncovered conserved biological pathways, such as translation regulation, ribosomal assembly, and RNA binding, suggesting a common regulatory role of m^5^C in breast cancer. Additionally, KEGG pathway analysis of methylated transcripts in breast cancer cell lines revealed consistent enrichment of pathways linked to cancer metabolism, protein processing, and DNA repair. Central carbon metabolism, glycolysis, and the TCA cycle were prominent, reflecting extensive metabolic reprogramming of breast cancer cells to support growth. Protein processing in the endoplasmic reticulum was enriched across all lines, suggesting increased stress response and protein synthesis. The enrichment of DNA repair pathways in breast cancer reflects underlying genomic instability, highlighting their essential contribution to disease progression. Literature confirms the regulatory impact of m^5^C in these processes, supporting the functional relevance of our findings. For instance, high expression levels of NSUN2 have been related to RNA degradation, signaling, RNA polymerase and spliceosome (Tong et al. 2022; Yang et al. 2017). To the best of our knowledge, studies focused solely on m^5^C in breast cancer are still limited. Previous reports highlighted that cancer-associated pathways, including the ERK-MAPK and PI3K-AKT, are activated as response to increased methylation levels in several mRNAs (Huang et al. 2021; Samatar and Poulikakos 2014; Sun et al. 2001). A recent study focused on TNBC suggests that m^5^C regulators are abnormally expressed, indicating a key role of m^5^C-machinery in both pathogenesis and development of aggressive breast cancer phenotypes (Huang et al. 2021). This finding is in line with previous studies that highlight the overexpression of NSUN2 and its role as a tumor-promoting factor in breast cancer, enhancing cell proliferation, migration, and invasion (Yang et al. 2017). Our results challenge this notion indicating a potential multifaceted role of NSUN2-mediated m^5^C methylation in breast cancer development and progression.

In conclusion, our study provides the first comprehensive analysis of the m⁵C epitranscriptome across the spectrum of different breast cancer molecular subtypes. The findings underscore the importance of m^5^C modifications in post-transcriptional gene regulation and offer new perspectives for understanding the molecular mechanisms underlying breast cancer. Future studies focusing on the dynamic regulation of m^5^C and its interaction with other RNA modifications will further elucidate the precise biological mechanisms by which m^5^C modifications are implicated in breast cancer biology and enhance our understanding of the epitranscriptomic landscape in cancer, unraveling novel therapeutic opportunities.

## Electronic supplementary material

Below is the link to the electronic supplementary material.

Supplementary Material 1

Supplementary Material 2

Supplementary Material 3

Supplementary Material 4

## Data Availability

The datasets supporting the findings of this study are available on request from the corresponding author.
