# Supplementary material for: Exploring the m5C epitranscriptome of mRNAs in breast cancer cells through genome engineering and long-read sequencing approaches
Source: Funct Integr Genomics. 2025 Jun 25;25(1):136. doi: 10.1007/s10142-025-01648-4 (PMC12187793; doi:10.1007/s10142-025-01648-4)

Gene Ontology (GO) enrichment analysis

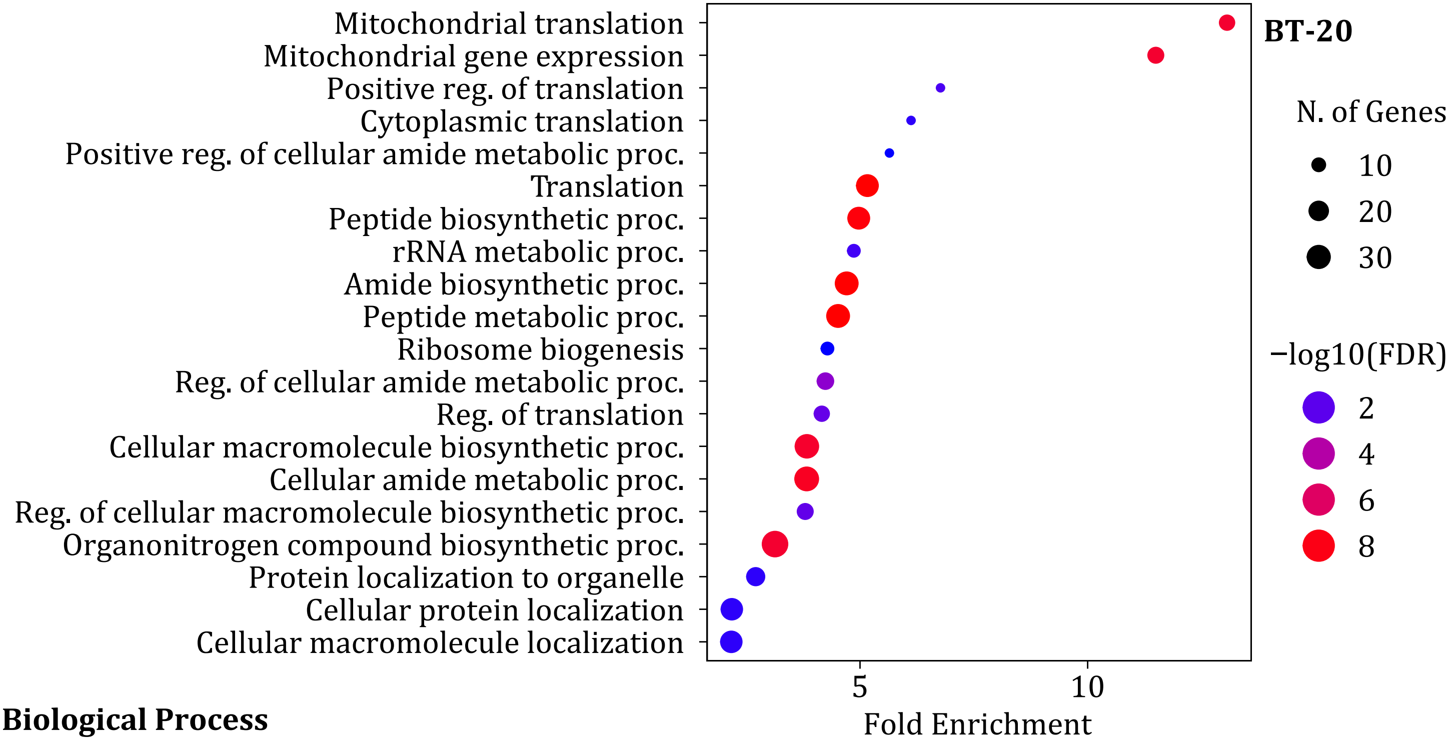
Mitochondrial translation
Mitochondrial gene expression
Positive reg. of translation
Cytoplasmic translation
Positive reg. of cellular amide metabolic proc.
Translation
Peptide biosynthetic proc.
rRNA metabolic proc.
Amide biosynthetic proc.
Peptide metabolic proc.
Ribosome biogenesis
Reg. of cellular amide metabolic proc.
Reg. of translation
Cellular macromolecule biosynthetic proc.
Cellular amide metabolic proc.
Reg. of cellular macromolecule biosynthetic proc.
Organonitrogen compound biosynthetic proc.
Protein localization to organelle
Cellular protein localization
Cellular macromolecule localization
5
10
Fold Enrichment
BT-20
N. of Genes
10
20
30
−log10(FDR)
2
4
6
8
Biological Process

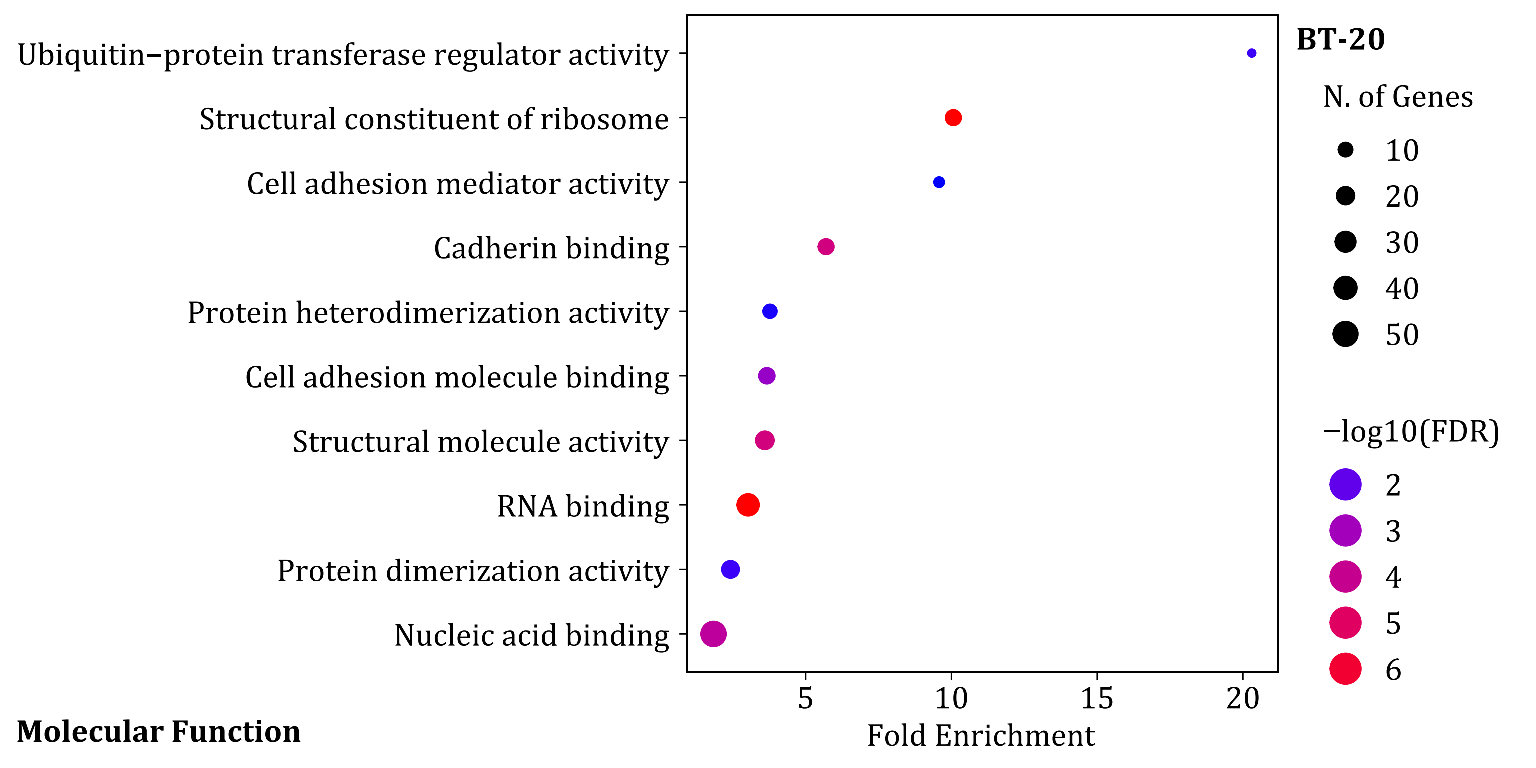
Ubiquitin−protein transferase regulator activity
Structural constituent of ribosome
Cell adhesion mediator activity
Cadherin binding
Protein heterodimerization activity
Cell adhesion molecule binding
Structural molecule activity
RNA binding
Protein dimerization activity
Nucleic acid binding
5
10
15
20
Fold Enrichment
BT-20
N. of Genes
10
20
30
40
50
−log10(FDR)
2
3
4
5
6
Molecular Function

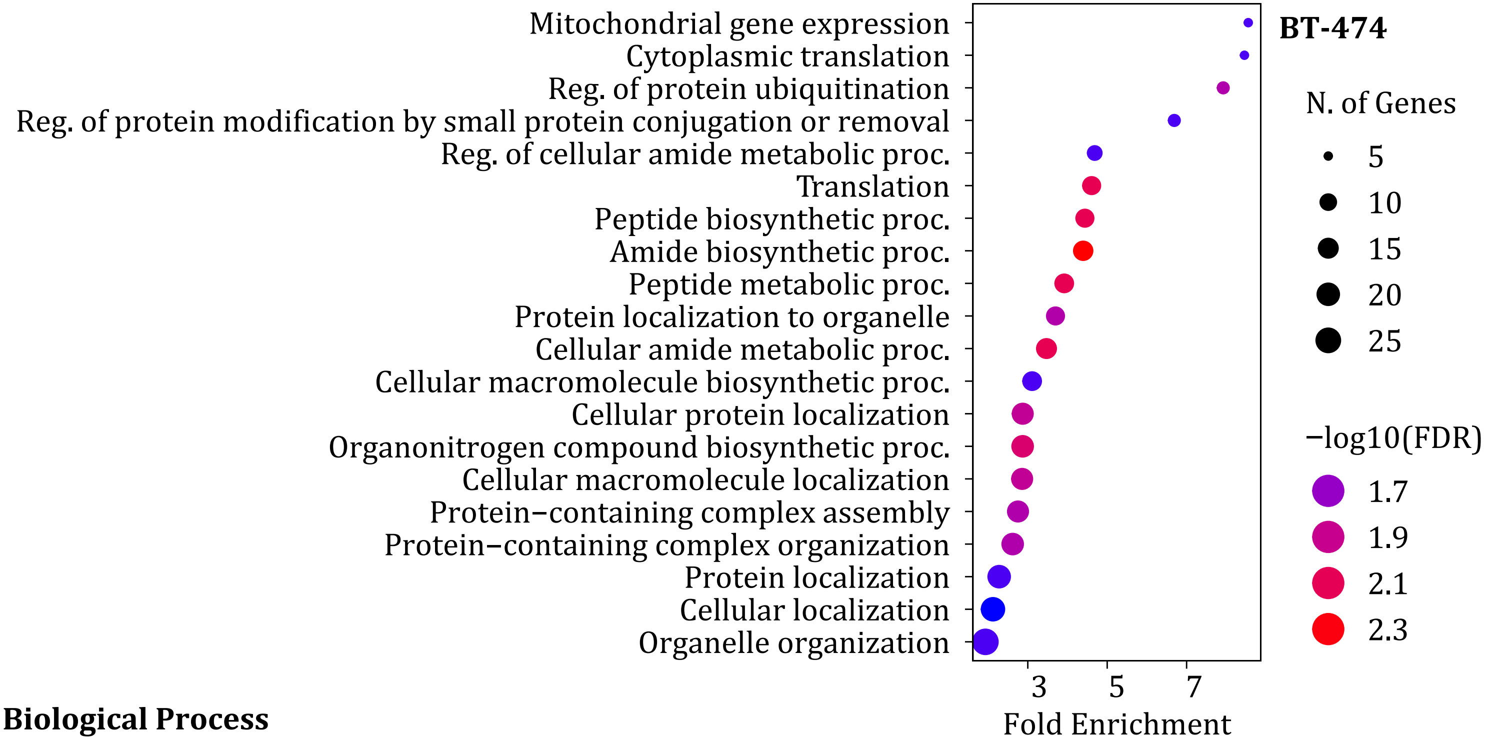
BT-474
Mitochondrial gene expression
Cytoplasmic translation
Reg. of protein ubiquitination
Reg. of protein modification by small protein conjugation or removal
Reg. of cellular amide metabolic proc.
Translation
Peptide biosynthetic proc.
Amide biosynthetic proc.
Peptide metabolic proc.
Protein localization to organelle
Cellular amide metabolic proc.
Cellular macromolecule biosynthetic proc.
Cellular protein localization
Organonitrogen compound biosynthetic proc.
Cellular macromolecule localization
Protein−containing complex assembly
Protein−containing complex organization
Protein localization
Cellular localization
Organelle organization
3
5
7
Fold Enrichment
N. of Genes
5
10
15
20
25
−log10(FDR)
1.7
1.9
2.1
2.3
Biological Process

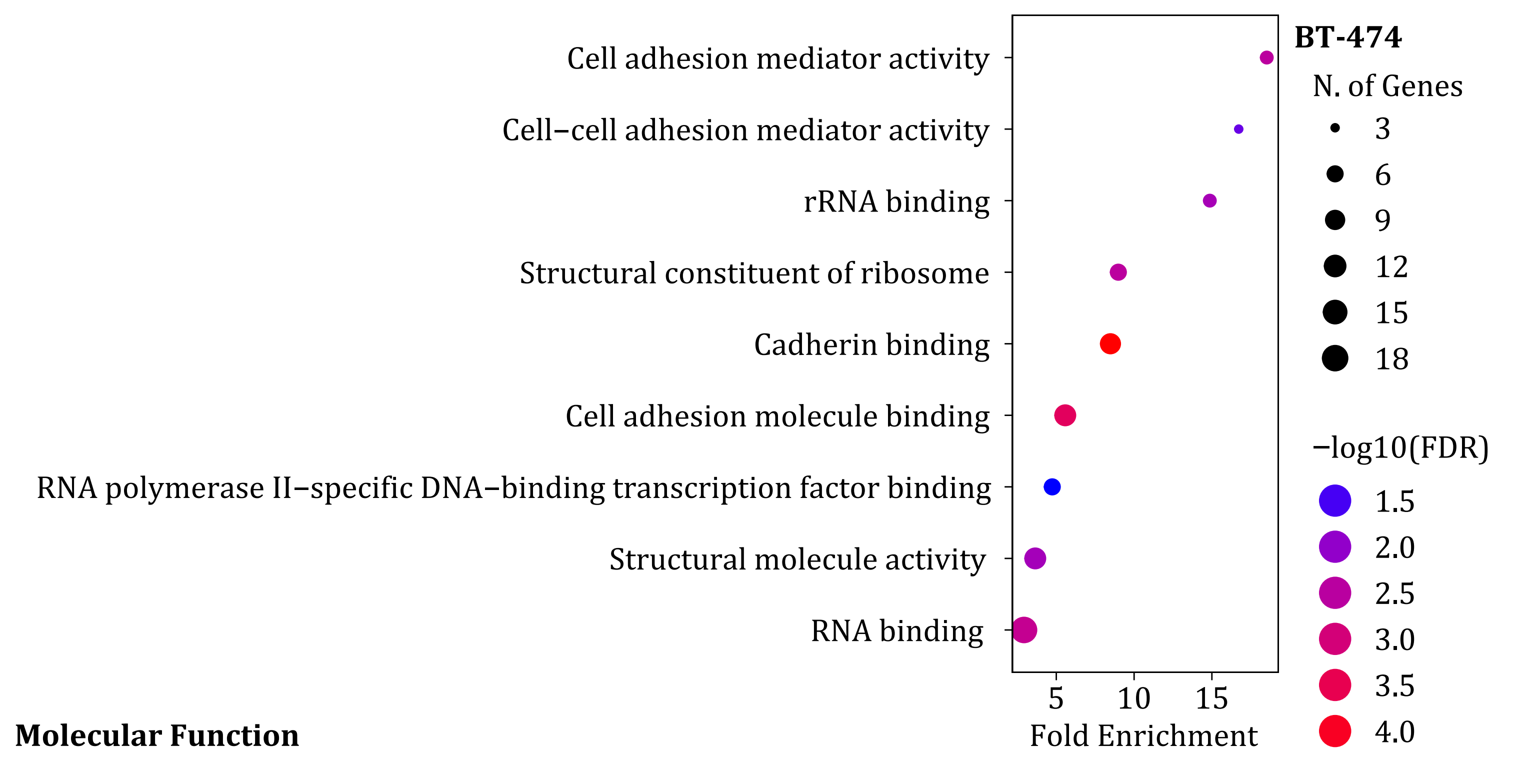
BT-474
Cell adhesion mediator activity
Cell−cell adhesion mediator activity
rRNA binding
Structural constituent of ribosome
Cadherin binding
Cell adhesion molecule binding
RNA polymerase II−specific DNA−binding transcription factor binding
Structural molecule activity
RNA binding
5
10
15
Fold Enrichment
N. of Genes
3
6
9
12
15
18
−log10(FDR)
1.5
2.0
2.5
3.0
3.5
4.0
Molecular Function

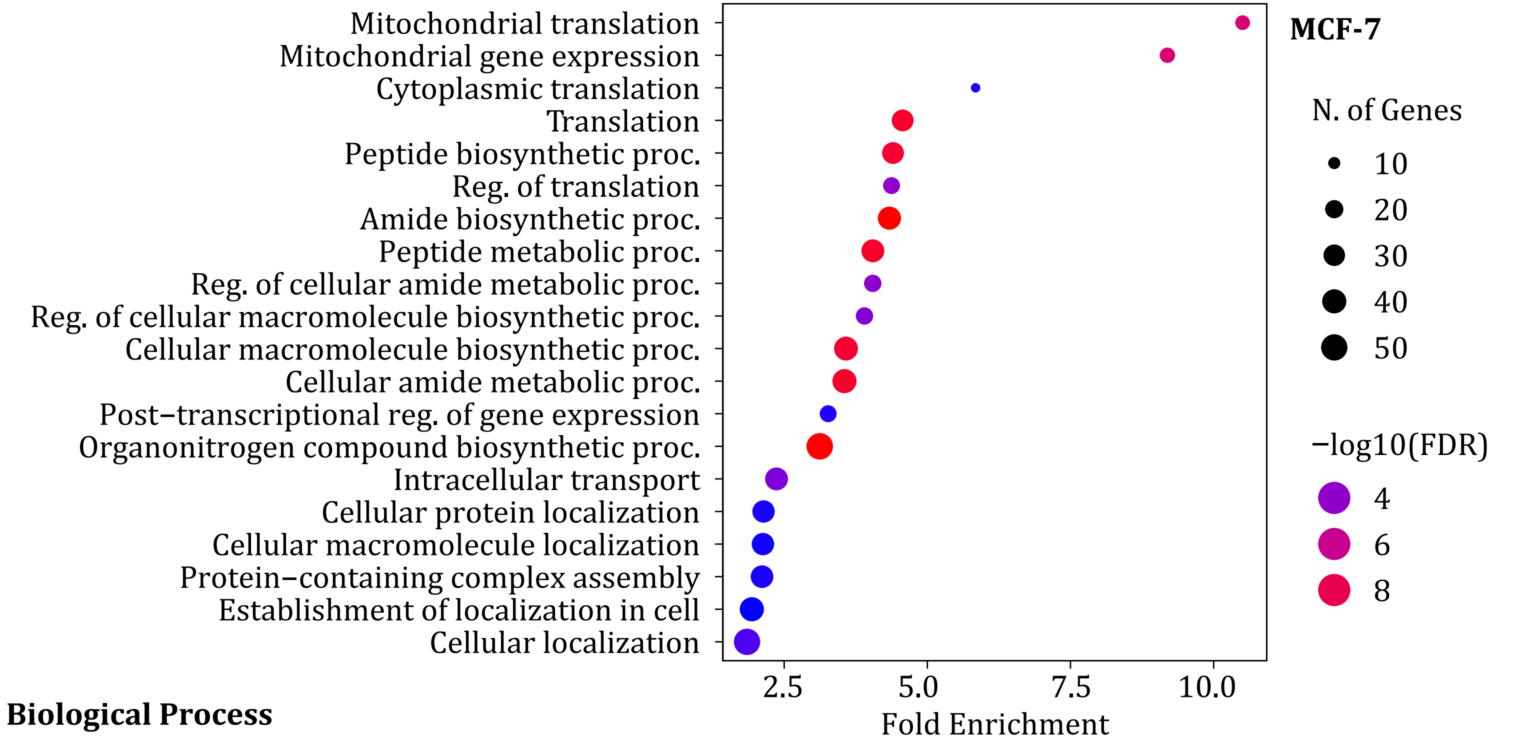

Mitochondrial translation
Mitochondrial gene expression
Cytoplasmic translation
Translation
Peptide biosynthetic proc.
Reg. of translation
Amide biosynthetic proc.
Peptide metabolic proc.
Reg. of cellular amide metabolic proc.
Reg. of cellular macromolecule biosynthetic proc.
Cellular macromolecule biosynthetic proc.
Cellular amide metabolic proc.
Post−transcriptional reg. of gene expression
Organonitrogen compound biosynthetic proc.
Intracellular transport
Cellular protein localization
Cellular macromolecule localization
Protein−containing complex assembly
Establishment of localization in cell
Cellular localization
2.5
5.0
7.5
10.0
Fold Enrichment
MCF-7
N. of Genes
10
20
30
40
50
−log10(FDR)
4
6
8
Biological Process

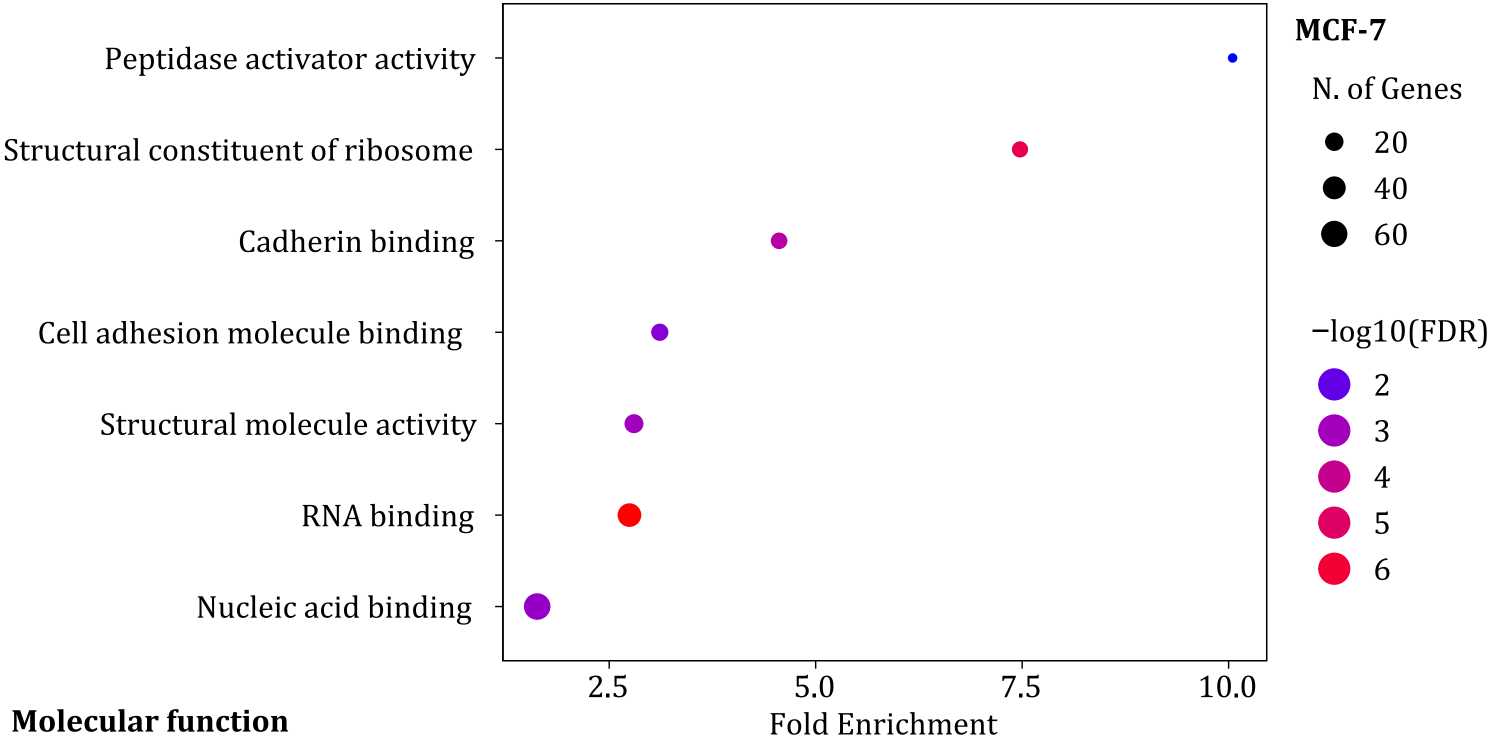

Peptidase activator activity
Structural constituent of ribosome
Cadherin binding
Cell adhesion molecule binding
Structural molecule activity
RNA binding
Nucleic acid binding
2.5
5.0
7.5
10.0
Fold Enrichment
MCF-7
N. of Genes
20
40
60
−log10(FDR)
2
3
4
5
6
Molecular function

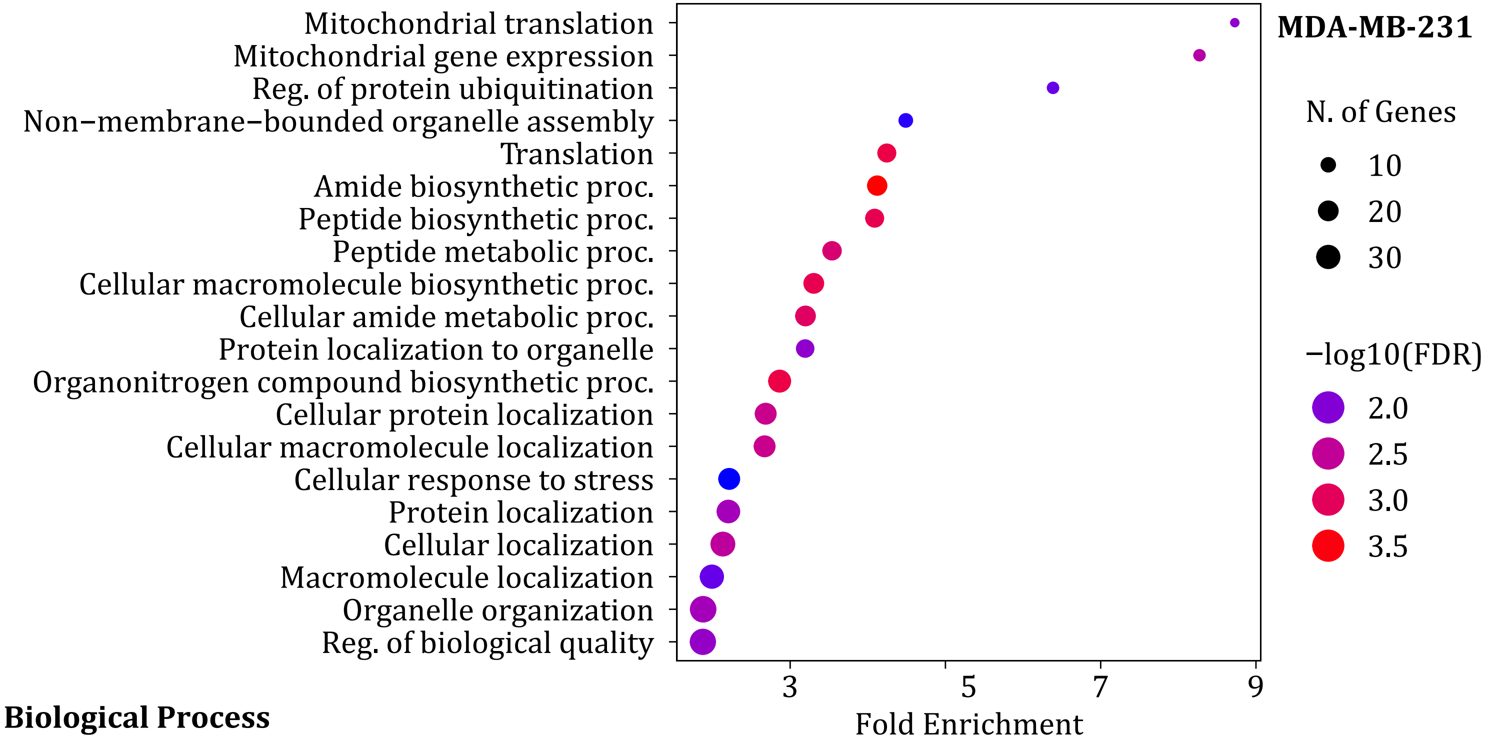

MDA-MB-231
Mitochondrial translation
Mitochondrial gene expression
Reg. of protein ubiquitination
Non−membrane−bounded organelle assembly
Translation
Amide biosynthetic proc.
Peptide biosynthetic proc.
Peptide metabolic proc.
Cellular macromolecule biosynthetic proc.
Cellular amide metabolic proc.
Protein localization to organelle
Organonitrogen compound biosynthetic proc.
Cellular protein localization
Cellular macromolecule localization
Cellular response to stress
Protein localization
Cellular localization
Macromolecule localization
Organelle organization
Reg. of biological quality
3
5
7
9
Fold Enrichment
N. of Genes
10
20
30
−log10(FDR)
2.0
2.5
3.0
3.5
Biological Process

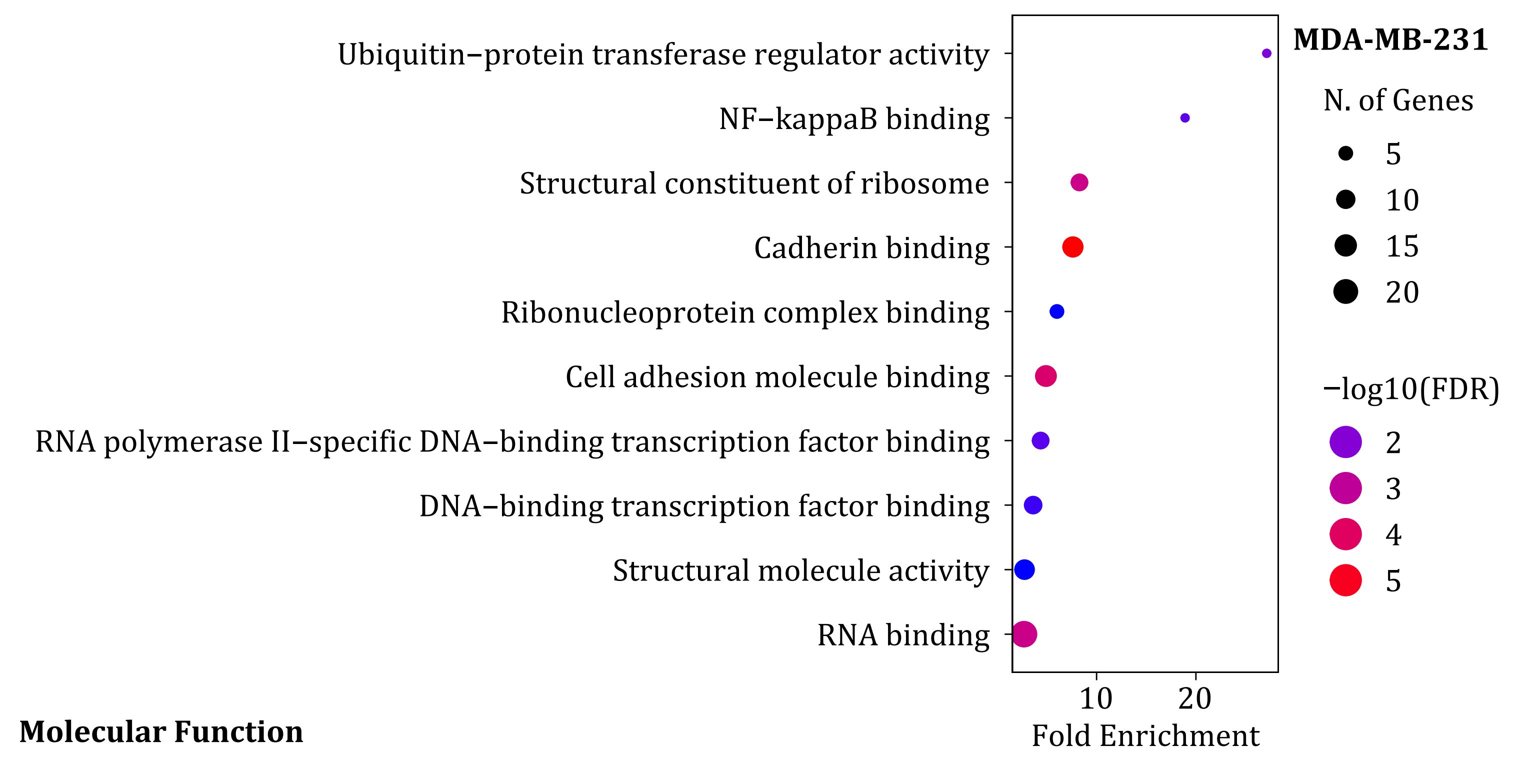

MDA-MB-231
Ubiquitin−protein transferase regulator activity
NF−kappaB binding
Structural constituent of ribosome
Cadherin binding
Ribonucleoprotein complex binding
Cell adhesion molecule binding
RNA polymerase II−specific DNA−binding transcription factor binding
DNA−binding transcription factor binding
Structural molecule activity
RNA binding
10
20
Fold Enrichment
N. of Genes
5
10
15
20
−log10(FDR)
2
3
4
5
Molecular Function

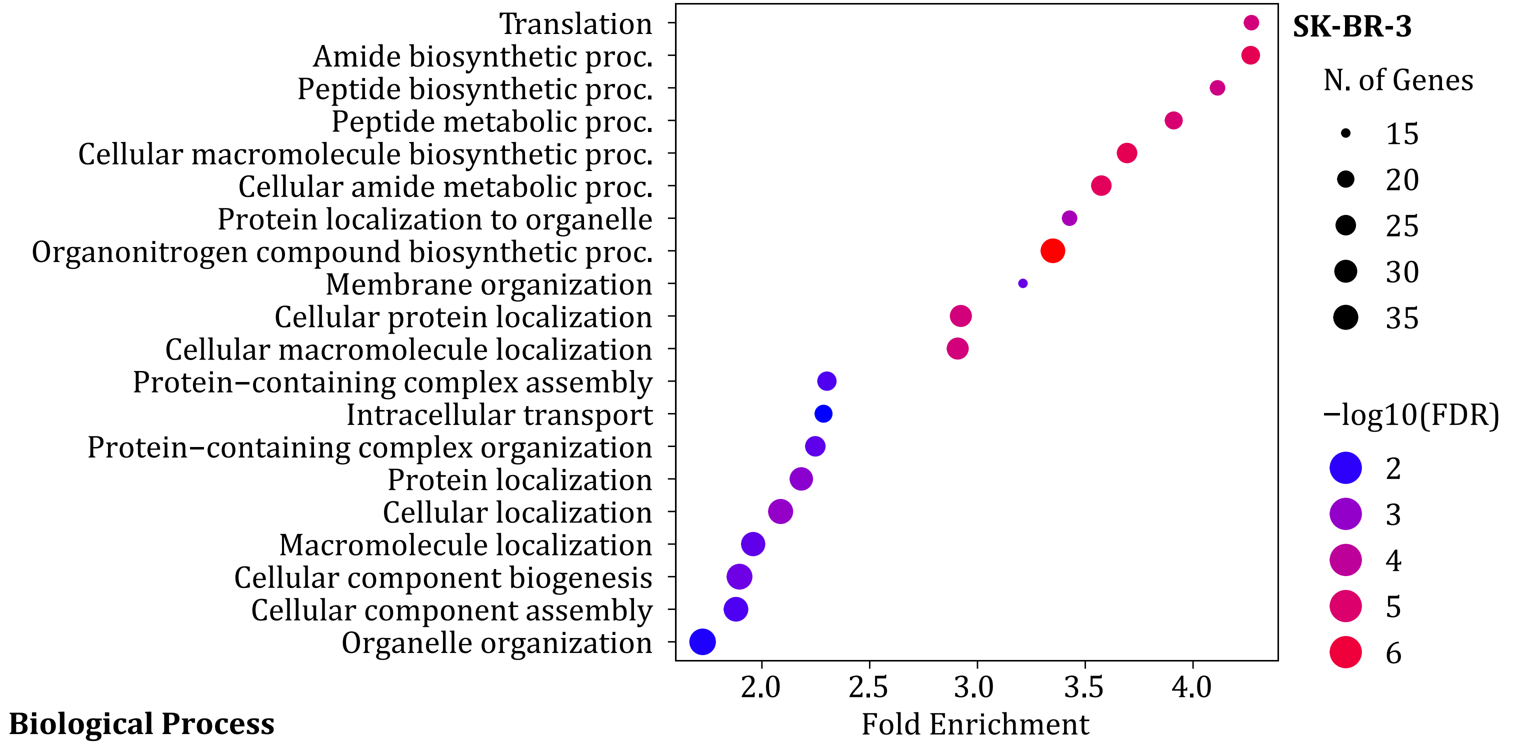

Translation
Amide biosynthetic proc.
Peptide biosynthetic proc.
Peptide metabolic proc.
Cellular macromolecule biosynthetic proc.
Cellular amide metabolic proc.
Protein localization to organelle
Organonitrogen compound biosynthetic proc.
Membrane organization
Cellular protein localization
Cellular macromolecule localization
Protein−containing complex assembly
Intracellular transport
Protein−containing complex organization
Protein localization
Cellular localization
Macromolecule localization
Cellular component biogenesis
Cellular component assembly
Organelle organization
2.0
2.5
3.0
3.5
4.0
Fold Enrichment
SK-BR-3
N. of Genes
15
20
25
30
35
−log10(FDR)
2
3
4
5
6
Biological Process

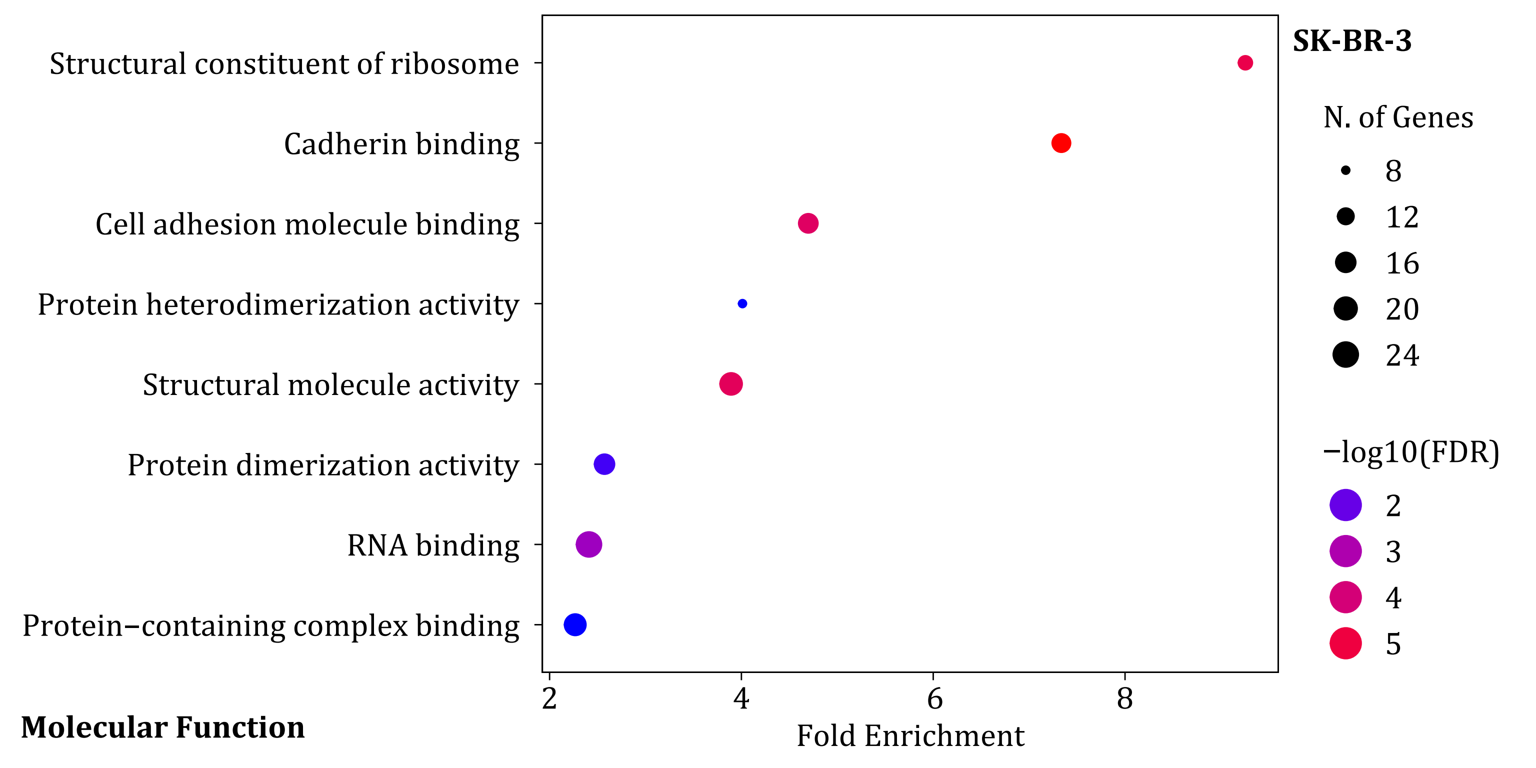

Structural constituent of ribosome
Cadherin binding
Cell adhesion molecule binding
Protein heterodimerization activity
Structural molecule activity
Protein dimerization activity
RNA binding
Protein−containing complex binding
2
4
6
8
Fold Enrichment
SK-BR-3
N. of Genes
8
12
16
20
24
−log10(FDR)
2
3
4
5
Molecular Function

KEGG pathway enrichment analysis

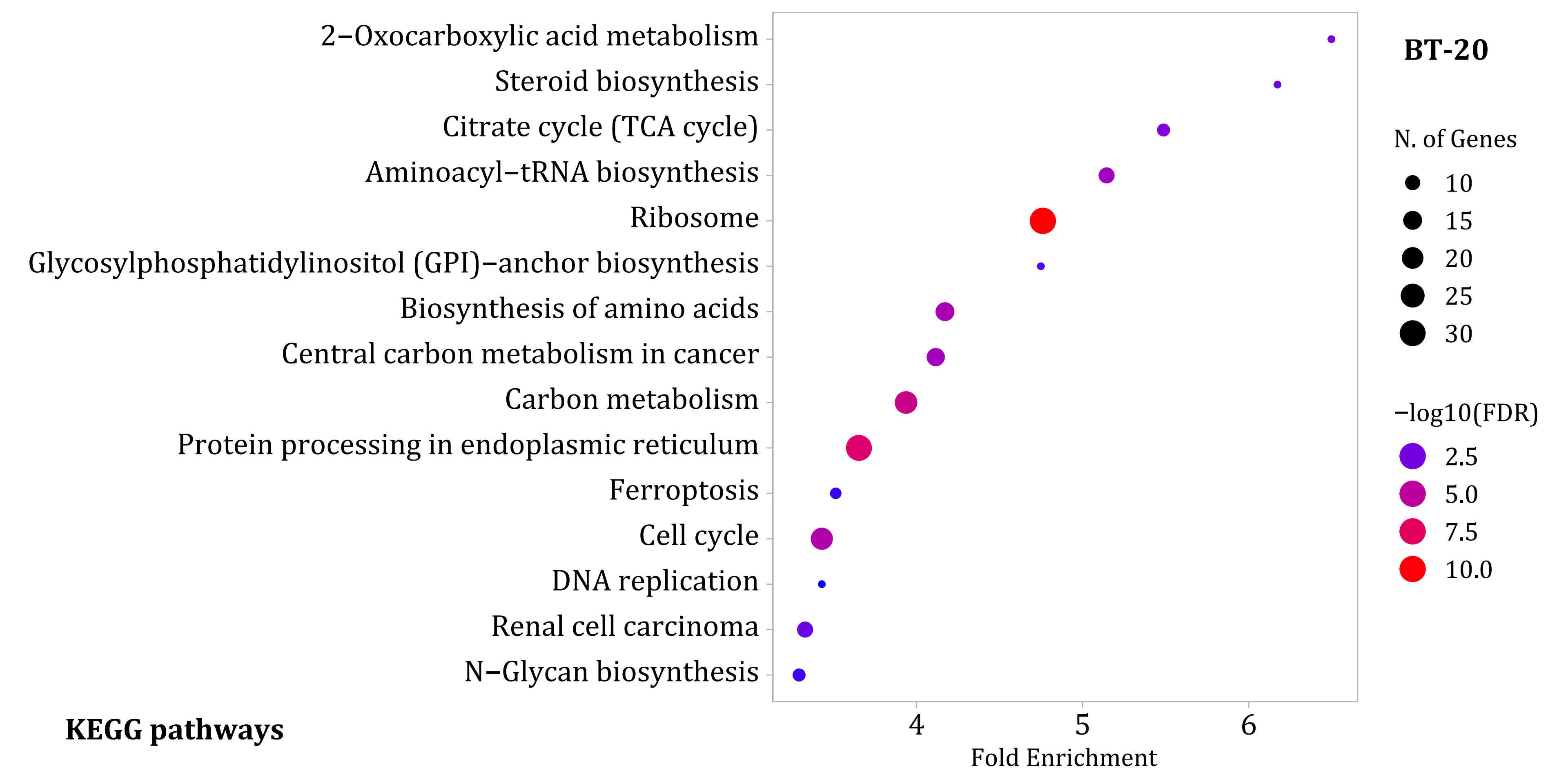

2−Oxocarboxylic acid metabolism
Steroid biosynthesis
Citrate cycle (TCA cycle)
Aminoacyl−tRNA biosynthesis
Ribosome
Glycosylphosphatidylinositol (GPI)−anchor biosynthesis
Biosynthesis of amino acids
Central carbon metabolism in cancer
Carbon metabolism
Protein processing in endoplasmic reticulum
Ferroptosis
Cell cycle
DNA replication
Renal cell carcinoma
N−Glycan biosynthesis
KEGG pathways
4
5
6
Fold Enrichment
BT-20
N. of Genes
10
15
20
25
30
−log10(FDR)
2.5
5.0
7.5
10.0

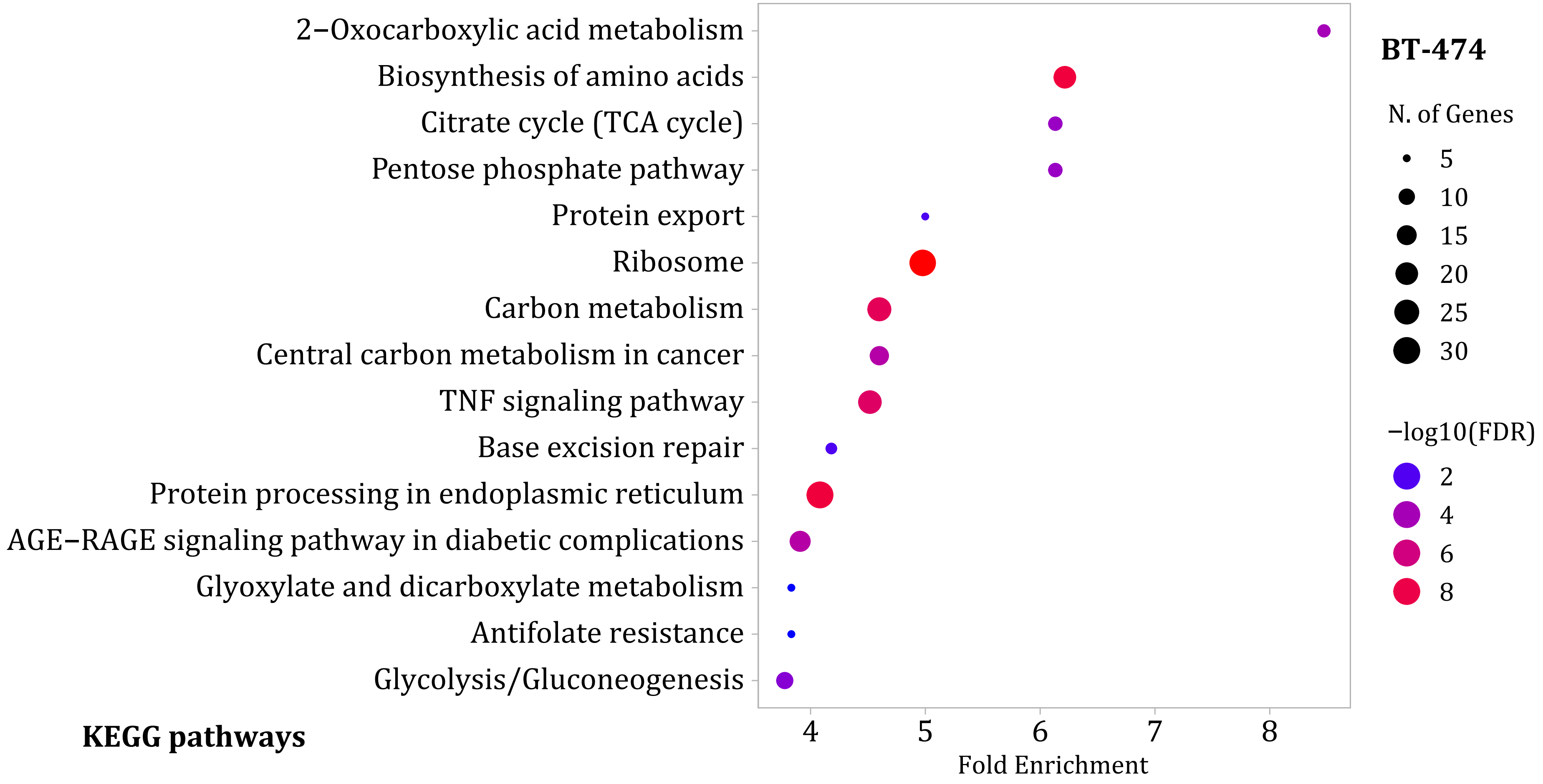

2−Oxocarboxylic acid metabolism
Biosynthesis of amino acids
Citrate cycle (TCA cycle)
Pentose phosphate pathway
Protein export
Ribosome
Carbon metabolism
Central carbon metabolism in cancer
TNF signaling pathway
Base excision repair
Protein processing in endoplasmic reticulum
AGE−RAGE signaling pathway in diabetic complications
Glyoxylate and dicarboxylate metabolism
Antifolate resistance
Glycolysis/Gluconeogenesis
KEGG pathways
4
5
6
7
8
Fold Enrichment
BT-474
N. of Genes
5
10
15
20
25
30
−log10(FDR)
2
4
6
8

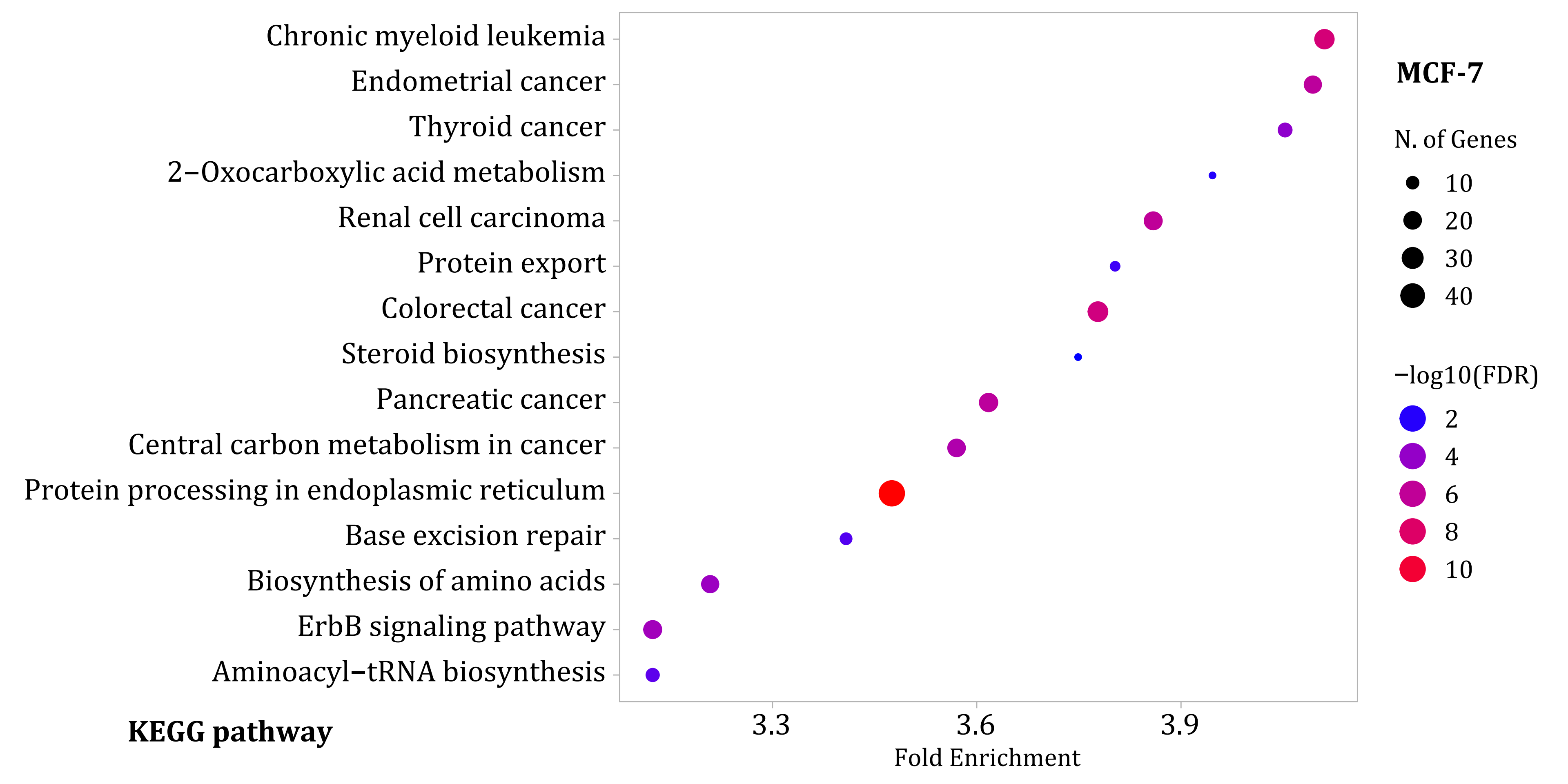

Chronic myeloid leukemia
Endometrial cancer
Thyroid cancer
2−Oxocarboxylic acid metabolism
Renal cell carcinoma
Protein export
Colorectal cancer
Steroid biosynthesis
Pancreatic cancer
Central carbon metabolism in cancer
Protein processing in endoplasmic reticulum
Base excision repair
Biosynthesis of amino acids
ErbB signaling pathway
Aminoacyl−tRNA biosynthesis
KEGG pathway
3.3
3.6
3.9
Fold Enrichment
MCF-7
N. of Genes
10
20
30
40
−log10(FDR)
2
4
6
8
10

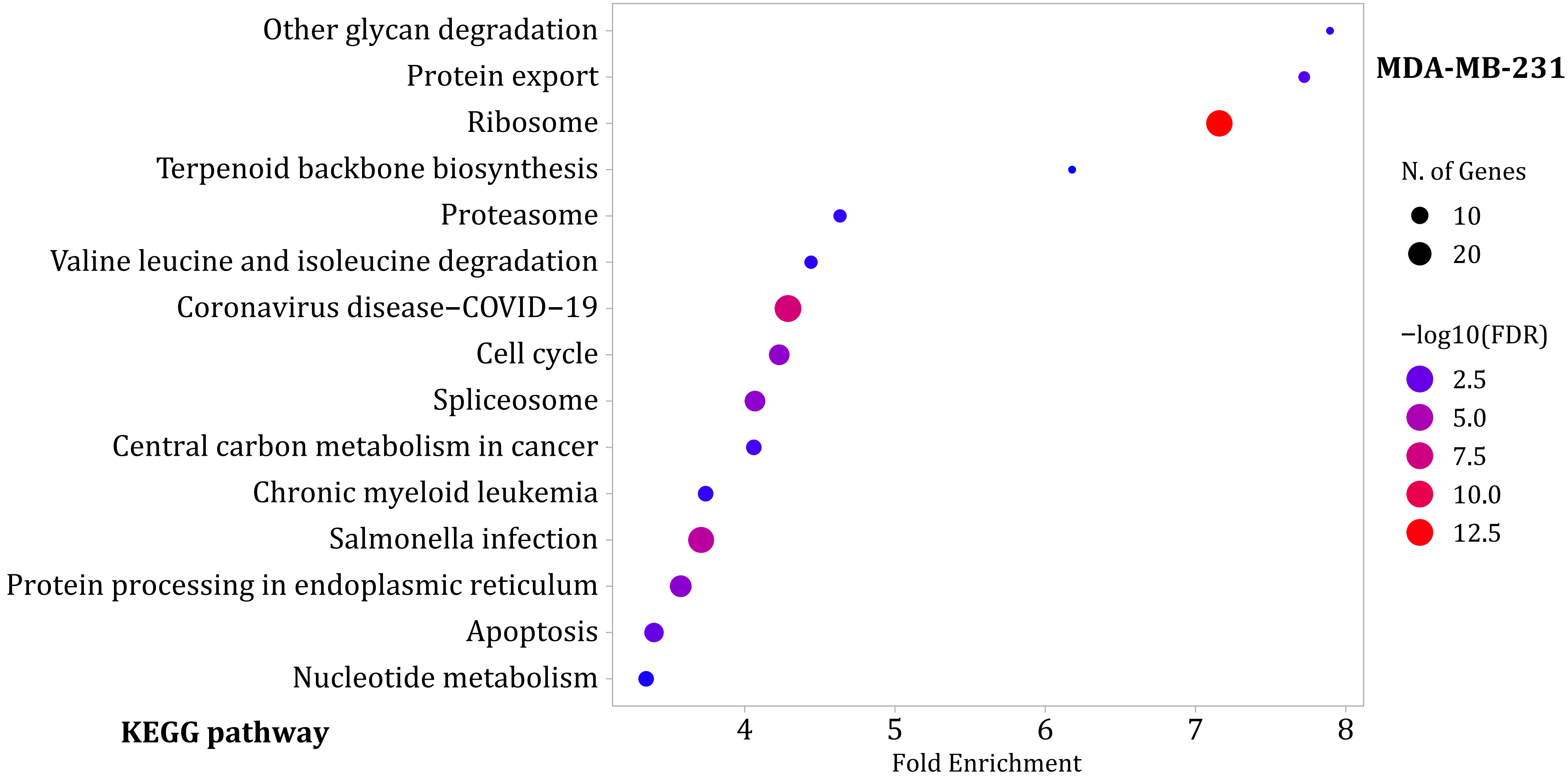

Other glycan degradation
Protein export
Ribosome
Terpenoid backbone biosynthesis
Proteasome
Valine leucine and isoleucine degradation
Coronavirus disease−COVID−19
Cell cycle
Spliceosome
Central carbon metabolism in cancer
Chronic myeloid leukemia
Salmonella infection
Protein processing in endoplasmic reticulum
Apoptosis
Nucleotide metabolism
KEGG pathway
4
5
6
7
8
Fold Enrichment
MDA-MB-231
N. of Genes
10
20
−log10(FDR)
2.5
5.0
7.5
10.0
12.5

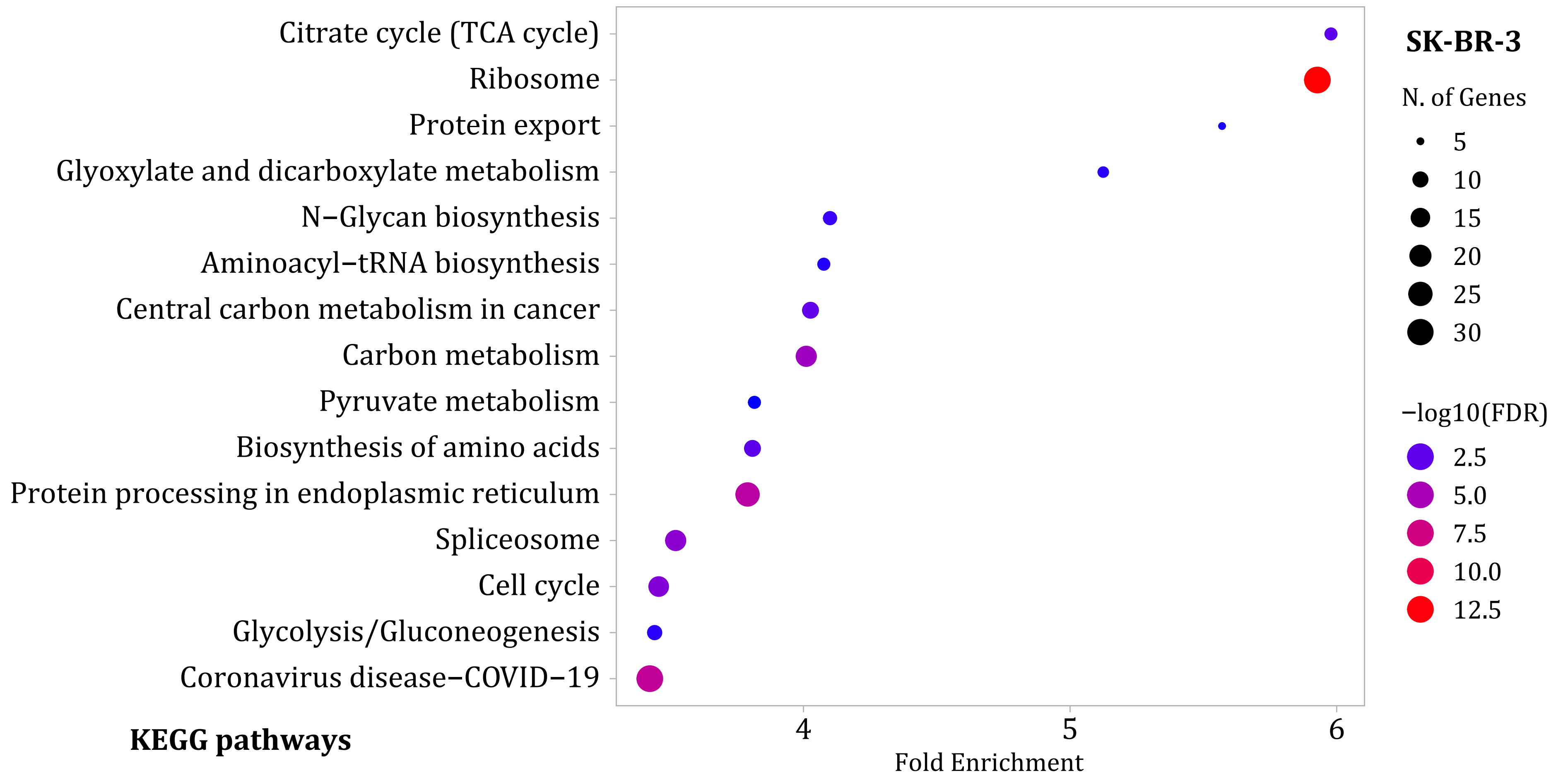

Citrate cycle (TCA cycle)
Ribosome
Protein export
Glyoxylate and dicarboxylate metabolism
N−Glycan biosynthesis
Aminoacyl−tRNA biosynthesis
Central carbon metabolism in cancer
Carbon metabolism
Pyruvate metabolism
Biosynthesis of amino acids
Protein processing in endoplasmic reticulum
Spliceosome
Cell cycle
Glycolysis/Gluconeogenesis
Coronavirus disease−COVID−19
KEGG pathways
4
5
6
Fold Enrichment
SK-BR-3
N. of Genes
5
10
15
20
25
30
−log10(FDR)
2.5
5.0
7.5
10.0
12.5

Full Western Blot images

NSUN2 antibody (#44056)

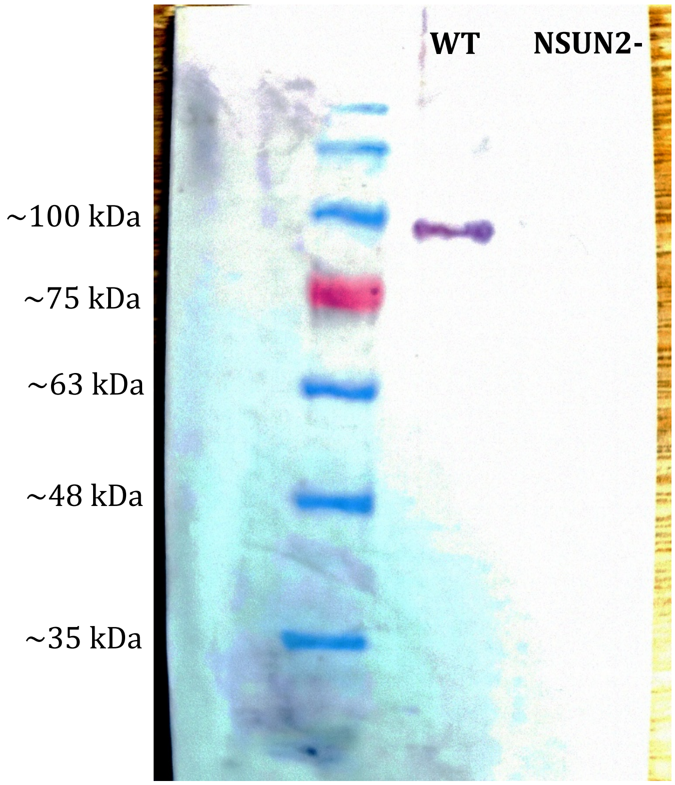

ACTB antibody (#4967)

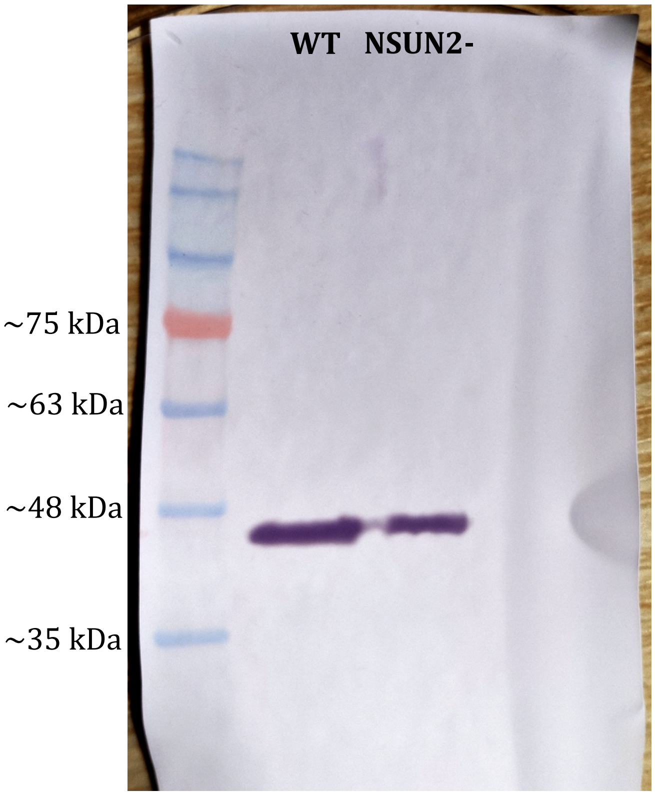

Supplement: Supplementary file 1 — Supplementary Material 1 [file 10142_2025_1648_MOESM1_ESM.pdf]
