# Supplementary material for: Exploring the m5C epitranscriptome of mRNAs in breast cancer cells through genome engineering and long-read sequencing approaches
Source: Funct Integr Genomics. 2025 Jun 25;25(1):136. doi: 10.1007/s10142-025-01648-4 (PMC12187793; doi:10.1007/s10142-025-01648-4)
Supplement: Supplementary file 2 — Supplementary Material 2 [file 10142_2025_1648_MOESM2_ESM.docx]

**Supplementary Table S1.** List of primers that were used for qPCR expression analysis of the canonical *NSUN2* targets. The melting temperature (T_m_) for each primer was calculated by Primer-BLAST designing tool.

| **Target** | **Primers** | | | | |
| --- | --- | --- | --- | --- | --- |
|  | **Direction** | **Sequence (5′→3′)** | **Length (nt)** | **Tm (^o^C)** | **Amplicon (bp)** |
| *ALYREF* | Forward | TTTGGTGGTGGTGGAGGC | 18 | 59.8 | 168 |
|  | Reverse | GCGGATTTGCTGGTCTGTTTA | 21 | 59.19 |  |
| *YBX1* | Forward | ATGCAGCAGACCGTAACCAT | 20 | 59.75 | 230 |
|  | Reverse | CTGCACAGGAGGGTTGGAAT | 20 | 59.96 |  |
| *TP53* | Forward | AAGGGTCAGTCTACCTCCCG | 20 | 60.32 | 193 |
|  | Reverse | GGAAGTCCTGGGTGCTTCTG | 20 | 60.32 |  |
| *TRMT112* | Forward | TGCCCGGAATCTGGACGTAT | 20 | 61.05 | 194 |
|  | Reverse | GTGTTTGGTGTCATTGGGTCA | 21 | 58.97 |  |
| *NOP2* | Forward | ACAGCTGGTAAGAAGGGACC | 20 | 59.02 | 196 |
|  | Reverse | CATCCTCAGAGTTGGAGTCAGC | 22 | 60.42 |  |
| *DNMT2* | Forward | TCCACAAAAATATGCAATGGATGTA | 25 | 57.58 | 294 |
|  | Reverse | ACGGACCTTCTACAAGTGGG | 20 | 59.03 |  |
| *FBL* | Forward | GCGTAATGGAGGACACTTTGTG | 22 | 59.84 | 160 |
|  | Reverse | GGCATGGTCTCTTTCATATGGC | 22 | 59.44 |  |
| *LIN28A* | Forward | TCATCATGCCAAGGAATGCAA | 21 | 58.54 | 204 |
|  | Reverse | CCCATTGTGGCTCAATTCTGT | 21 | 58.83 |  |
| *IGF2BP1* | Forward | CAGAAGGGACAGAGTAACCAGG | 22 | 59.77 | 174 |
|  | Reverse | AGTGGGCAAACCTGATCTACAG | 22 | 60.03 |  |
