# Supplementary material for: Exploring the m5C epitranscriptome of mRNAs in breast cancer cells through genome engineering and long-read sequencing approaches
Source: Funct Integr Genomics. 2025 Jun 25;25(1):136. doi: 10.1007/s10142-025-01648-4 (PMC12187793; doi:10.1007/s10142-025-01648-4)
Supplement: Supplementary file 3 — Supplementary Material 3 [file 10142_2025_1648_MOESM3_ESM.docx]

**Supplementary Table S2.** List of primers that were used for mRNA stability experiments. The melting temperature (T_m_) for each primer was calculated by Primer-BLAST designing tool.

| **Target** | **Primers** | | | | |  |
| --- | --- | --- | --- | --- | --- | --- |
|  | **Direction** | **Sequence (5′→3′)** | **Length (nt)** | **Tm (^o^C)** | **Amplicon (bp)** | |
| *ELF3* | Forward | GCTCTTCCCCAGCGATGGTT | 20 | 62.54 | 122 | |
|  | Reverse | TCTTCTTGCCCTCGAGACAGT | 21 | 60.82 |  |  |
| *DYNLL1* | Forward | GCCGTGATCAAAAATGCGGA | 20 | 59.83 | 106 | |
|  | Reverse | GAGCCGCAATGTCCTTCTCT | 20 | 60.11 |  |  |
| *HYLS1* | Forward | TTACCCACATCTGTGCAGGG | 20 | 59.67 | 193 | |
|  | Reverse | TTTCGGAGTCTTTGGGAGGC | 20 | 59.96 |  |  |
| *JUNB* | Forward | CTACTTTTCTGGTCAGGGCTCG | 22 | 60.68 | 112 | |
|  | Reverse | TGTAGGCGTCGTCGTGATCA | 20 | 61.30 |  |  |
| *NTHL1* | Forward | CAACAGGCTGAGGTGGACCA | 20 | 62.06 | 106 | |
|  | Reverse | GAGTCCATTGATCTCGTGCCA | 21 | 60.13 |  |  |
| *PUS1* | Forward | CCAAGAACAGATGTGATGCCAG | 22 | 59.58 | 227 | |
|  | Reverse | GTACATCTCCAGGATGTAGCG | 21 | 57.71 |  |  |
